# Innate immune sensor NOD2 promotes cartilage degradation and osteoarthritis progression by stabilizing TRAF6 in chondrocytes

**DOI:** 10.1172/jci.insight.196750

**Published:** 2026-08-24

**Authors:** Yuting Wang, Song Li, Yonghui Dong, Jiaming Zhang, Jian Liu, Zhenggang Wang, Shuang Liang, Nathan R. Martinez, Hongxu Pu, Peng Cheng, Anmin Chen, Qing Yang, Charles K.F. Chan, Wen Jiang, Jun Xiao, Fengjing Guo, Liming Zhao

**Affiliations:** 1 Department of Orthopaedics, Tongji Hospital, Tongji Medical College, Huazhong University of Science and Technology, Wuhan, Hubei, China.; 2 Institute for Stem Cell Biology and Regenerative Medicine and; 3 Department of Surgery, Division of Plastic and Reconstructive Surgery, Stanford University School of Medicine, Stanford, California, USA.; 4 Department of Orthopedics, Henan Provincial People’s Hospital, People’s Hospital of Zhengzhou University, Zhengzhou, Henan, China.; 5 Clinical Innovation and Research Center, Shenzhen Hospital, Southern Medical University, Shenzhen, Guangdong, China.; 6 Department of Radiation Oncology, The University of Texas MD Anderson Cancer Center, Houston, Texas, USA.

**Keywords:** Bone biology, Inflammation, Cartilage, Cellular immune response, Osteoarthritis

## Abstract

Inflammation driven by the innate immune response plays a crucial role in osteoarthritis (OA) pathogenesis, yet the underlying mechanisms remain incompletely understood. Moreover, current antiinflammatory therapies primarily offer symptomatic relief without altering disease progression. Nucleotide-binding oligomerization domain 2 (NOD2) is an intracellular pattern recognition receptor that detects a broad range of microbial and damage-associated stimuli and has been implicated in several inflammatory conditions. In this study, we investigated the role of NOD2 in OA-associated inflammation and cartilage degradation. Elevated NOD2 expression was observed in both human and mouse osteoarthritic cartilage. Conditional KO of *Nod2* in chondrocytes suppressed inflammation-induced catabolic responses in vitro and protected against cartilage degradation in mouse OA models. Mechanistically, we identified tumor necrosis factor receptor–associated factor 6 (TRAF6) as a key downstream mediator through which NOD2 promotes chondrocyte catabolism. Furthermore, we showed that pharmacological inhibition of NOD2 using 2 independent small-molecule inhibitors significantly attenuated OA progression in vivo. Collectively, these findings establish NOD2 as a critical regulator of OA-associated inflammation and cartilage degradation, and they highlight its potential as a therapeutic target for disease-modifying OA treatment.

## Abstract 2

Blocking a key immune sensor, NOD2, in cartilage cells reduces joint damage in osteoarthritis, revealing a promising new approach for treating this common disease.

## Introduction

Osteoarthritis (OA) is the most prevalent joint disease and a leading cause of chronic pain and long-term disability in adults (1, 2). Hallmark pathological features of OA include synovial inflammation, progressive cartilage degradation, and subchondral bone remodeling (3). While traditionally viewed as a degenerative disease driven by mechanical wear and tear, increasing evidence suggests that OA pathogenesis involves active contributions from the entire joint (4). Inflammation in OA is typically low-grade and chronic, largely driven by the innate immune system, and it plays a critical role in disease onset and progression (5). Cartilage extracellular matrix (ECM) debris resulting from mechanical wear, intracellular alarmins released by stressed or senescent cells, and leakage of trauma-associated plasma proteins act as damage-associated molecular patterns (DAMPs), which activate innate immune cells such as macrophages and mast cells to trigger immune responses and sustain inflammation (6). However, current antiinflammatory treatments mainly contribute to symptom relief rather than disease modification, highlighting the need to better understand the inflammatory mechanisms driving OA (7, 8).

Nucleotide-binding oligomerization domain 2 (NOD2) is a cytosolic pattern recognition receptor of the NOD-like receptor (NLR) family within the innate immune system, classically known for recognizing pathogen-associated molecular patterns (PAMPs), particularly muramyl dipeptide (MDP) derived from bacterial peptidoglycans (9, 10). Upon activation, NOD2 undergoes oligomerization and recruits receptor-interacting protein kinase 2 (RIPK2), leading to activation of downstream NF-κB and mitogen-activated protein kinase (MAPK) signaling pathways (11, 12). NOD2 has been implicated in several inflammatory diseases, including Crohn’s disease, Blau syndrome, and early-onset sarcoidosis (13–15). More recently, NOD2 has also been shown to recognize DAMPs, thereby contributing to sterile inflammation in various disease contexts (16). The involvement of NOD2 in OA has been implicated by its elevated expression in osteoarthritic synovial tissue and the association of NOD2 gene variants with familial susceptibility to OA (17, 18). However, the specific role of NOD2 in cartilage degradation, and particularly the function of NOD2 beyond the innate immune system, remains poorly defined.

In this study, we investigated the function of NOD2 in OA pathogenesis using conditional KO (cKO) mouse models and evaluated the therapeutic potential of pharmacologically targeting NOD2 with small-molecule inhibitors. Furthermore, we examined the molecular mechanisms by which NOD2 modulates chondrocyte catabolism and contributes to cartilage degradation.

## Results

### NOD2 is activated in both human and mouse osteoarthritic cartilage.

**Figure 1:**
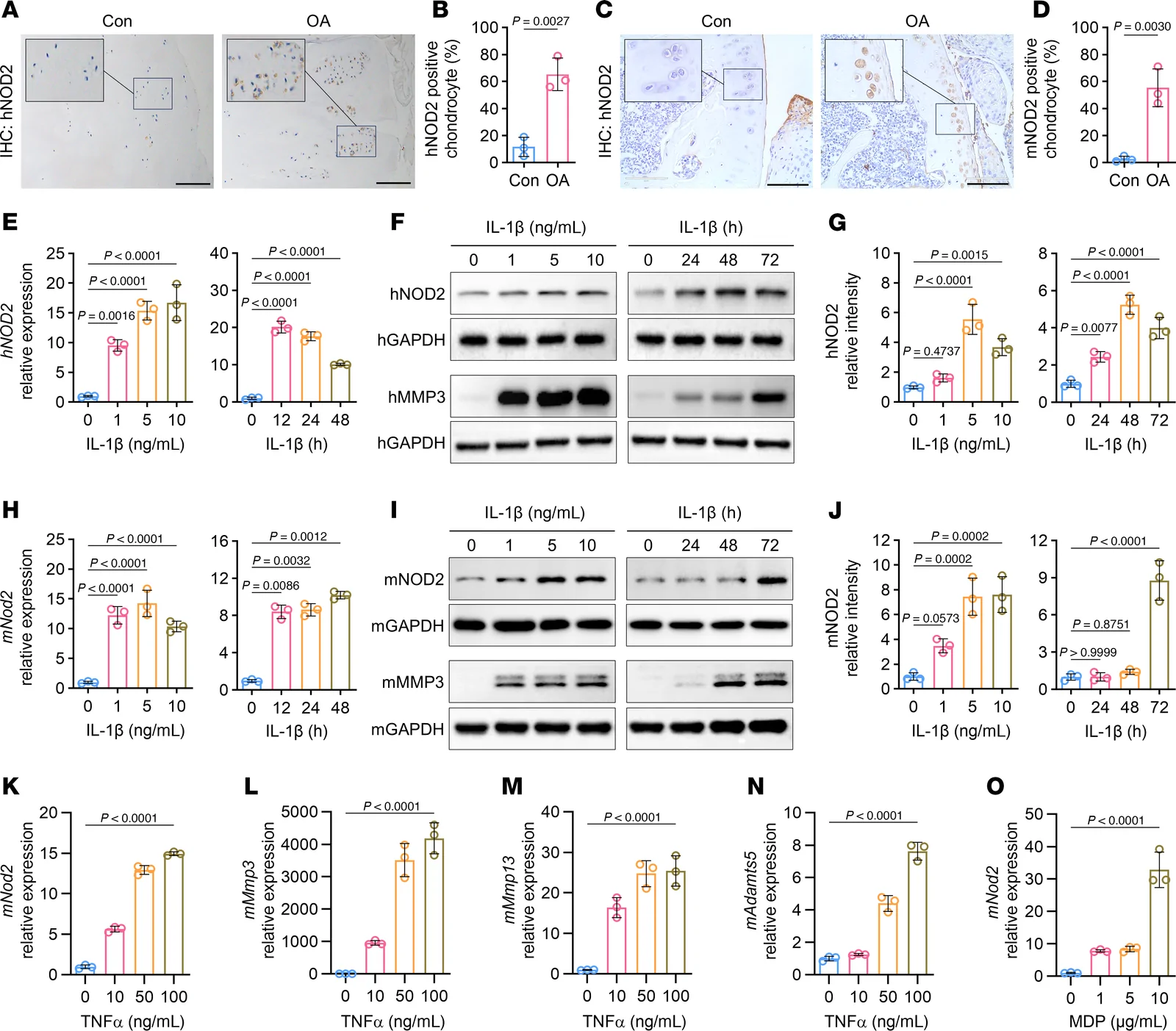
NOD2 is activated in both human and mouse osteoarthritic cartilage. (**A**) Representative IHC images of NOD2 in healthy and osteoarthritic human cartilage. Scale bar: 100 μm. (**B**) Quantification of NOD2^+^ chondrocytes. *n* = 3 donors/group. Two-tailed, unpaired Student’s *t* test. (**C**) Representative IHC images of NOD2 in healthy and osteoarthritic mouse cartilage. Scale bar: 50 μm. (**D**) Quantification of NOD2^+^ chondrocytes. *n* = 3 mice/group. Two-tailed, unpaired Student’s *t* test. (**E**) qPCR analysis of *NOD2* mRNA in primary human chondrocytes treated with IL-1β at indicated concentrations (for 24 h) and durations (using 5 ng/mL of IL-1β). *n* = 3 independent experiments. One-way ANOVA with Tukey’s multiple-comparison test. (**F** and **G**) Western blot analysis and densitometric quantification of NOD2 protein in primary human chondrocytes treated with IL-1β at indicated concentrations (for 48 h) and durations (using 5 ng/mL of IL-1β). *n* = 3 independent experiments. One-way ANOVA with Tukey’s multiple-comparison test. (**H**) qPCR analysis of *Nod2* mRNA in primary mouse chondrocytes treated with IL-1β at indicated concentrations (for 24 h) and durations (using 5 ng/mL of IL-1β). *n* = 3 independent experiments. One-way ANOVA with Tukey’s multiple-comparison test. (**I** and **J**) Western blot analysis and densitometric quantification of NOD2 protein in primary mouse chondrocytes treated with IL-1β at indicated concentrations (for 72 h) and durations (using 5 ng/mL of IL-1β). *n* = 3 independent experiments. One-way ANOVA with Tukey’s multiple-comparison test. (**K**–**N**) qPCR analysis of *Nod2*, *Mmp3*, *Mmp13*, and *ADAMTS5* mRNA levels in primary mouse chondrocytes treated with TNF-α at indicated concentrations (for 24 h). *n* = 3 independent experiments. One-way ANOVA with Tukey’s multiple-comparison test. (**O**) qPCR analysis of *Nod2* mRNA levels in primary mouse chondrocytes treated with muramyl dipeptide (MDP) at indicated concentrations (for 24 h). *n* = 3 independent experiments. One-way ANOVA with Tukey’s multiple-comparison test. Data are presented as mean ± SD.

To gain a more comprehensive understanding of its role in OA pathophysiology, we first examined NOD2 level in human and mouse osteoarthritic cartilage. Human osteoarthritic cartilage was obtained from patients undergoing total knee arthroplasty (Kellgren-Lawrence grade 3–4) (19), while healthy control cartilage was collected from donors undergoing amputation due to trauma or tumor (Kellgren-Lawrence grade 0). As summarized in Supplemental Table 1 (supplemental material available online with this article; https://doi.org/10.1172/jci.insight.196750DS1), demographic factors including age, sex, and BMI were comparable between the 2 groups. IHC staining of medial tibial plateau sections revealed significantly increased NOD2 protein level in osteoarthritic cartilage compared with healthy controls (65.33% ± 12.10% versus 11.67% ± 7.02%, *P* = 0.0027) (Figure 1, A and B). Similarly, in a mouse OA model induced by surgical destabilization of the medial meniscus (DMM) (20), NOD2 protein level was significantly elevated in articular cartilage (55.33% ± 14.01% versus 2.67% ± 2.08%, *P* = 0.0030) (Figure 1, C and D). Given that inflammatory cytokines such as IL-1β and TNF-α are key mediators of chondrocyte catabolism and cartilage degradation (22), we next investigated whether they regulate *NOD2* expression. In primary human chondrocytes, IL-1β stimulation induced a dose-dependent increase in *NOD2* expression at both RNA and protein levels, accompanied by upregulation of the catabolic enzyme matrix metalloproteinase-3 (*MMP3*). Peak mRNA and protein expression occurred at 12–24 hours and 48–72 hours after treatment, respectively (Figure 1, E–G). Consistently, IL-1β treatment also induced *Nod2* expression in primary mouse chondrocytes (Figure 1, H–J). In addition, TNF-α robustly upregulated both *Nod2* and catabolic enzyme expression (Figure 1, K–N). To confirm NOD2 activation, we treated mouse chondrocytes with MDP, a bacterial peptidoglycan-derived ligand known to activate NOD2 (22), and we observed increased *Nod2* expression (Figure 1O). These findings demonstrate that NOD2 expression is elevated in osteoarthritic cartilage and is inducible by proinflammatory stimuli, suggesting a potential role for NOD2 in chondrocyte catabolism and cartilage homeostasis during OA progression.

### Knockdown of Nod2 inhibits inflammatory cytokine-induced chondrocyte catabolism and apoptosis in vitro.

**Figure 2:**
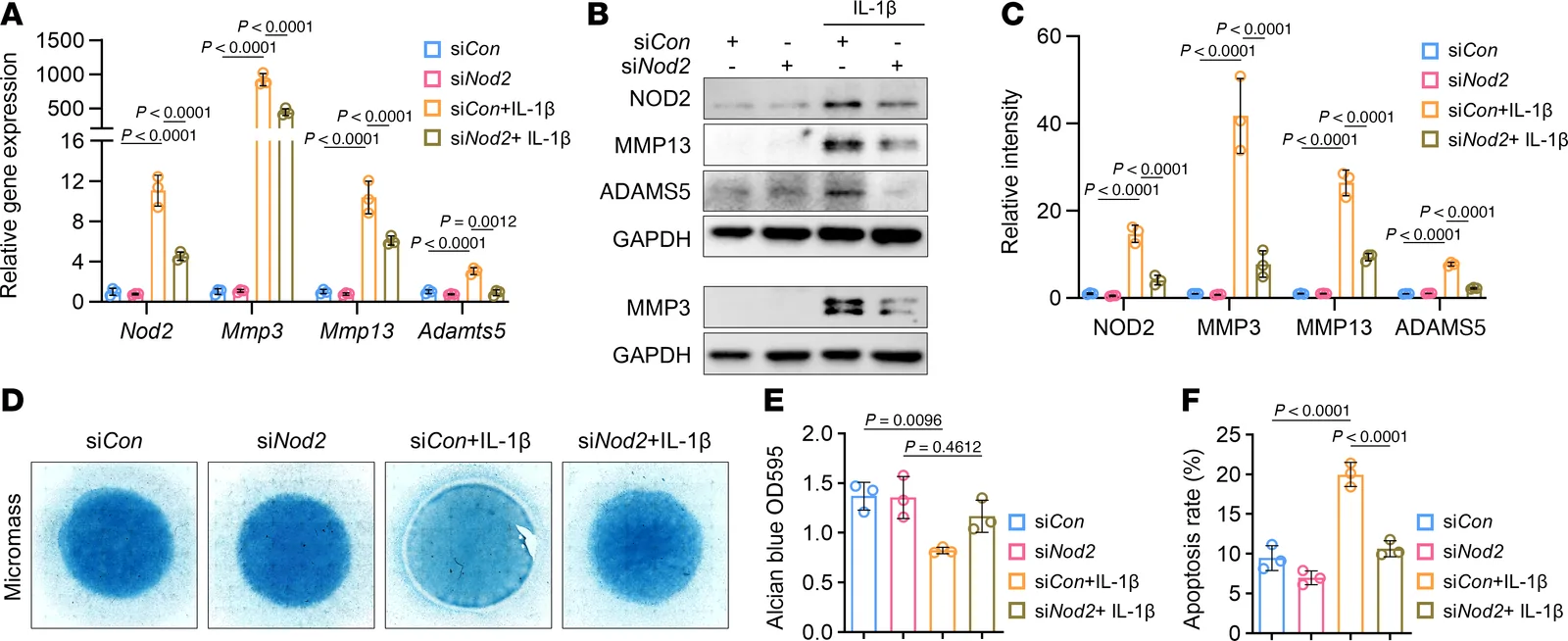
*Nod2* knockdown attenuates inflammation-induced chondrocyte catabolism and apoptosis in vitro. (**A**) qPCR analysis of *Nod2*, *Mmp3*, *Mmp13*, and *ADAMTS5* mRNA levels in primary mouse chondrocytes following *Nod2* knockdown and IL-1β treatment (5 ng/mL for 24 h). *n* = 3 independent experiments. One-way ANOVA with Tukey’s multiple-comparison test. (**B** and **C**) Western blot analysis and densitometric quantification of NOD2, MMP3, MMP13, and ADAMTS5 protein levels following *Nod2* knockdown and IL-1β treatment (5 ng/mL for 72 h). *n* = 3 independent experiments. One-way ANOVA with Tukey’s multiple-comparison test. (**D**) Alcian blue staining of micromass cultures of primary mouse chondrocytes following *Nod2* knockdown and IL-1β treatment (5 ng/mL for 2 weeks). (**E**) Quantification of proteoglycan content by absorbance of dissolved Alcian blue at 595 nm. *n* = 3 independent experiments. One-way ANOVA with Tukey’s multiple-comparison test. (**F**) Flow cytometric analysis of apoptosis in primary mouse chondrocytes following *Nod2* knockdown and IL-1β treatment (5 ng/mL for 24 h). *n* = 3 independent experiments. One-way ANOVA with Tukey’s multiple-comparison test. Data are presented as mean ± SD.

We next investigated whether NOD2 contributes to chondrocyte catabolism and apoptosis under inflammatory conditions. Small interfering RNA (siRNA) was used to knock down *Nod2* in primary mouse chondrocytes. As expected, IL-1β treatment induced catabolic activity, as indicated by increased mRNA and protein levels of matrix-degrading enzymes, including *Mmp3*, *Mmp13*, and *ADAMTS5*. However, *Nod2* knockdown significantly attenuated this inflammatory catabolic response (Figure 2, A–C). To further validate the protective effect of *Nod2* knockdown on cartilage matrix metabolism, we utilized a high-density micromass culture model of primary chondrocytes. Alcian blue staining demonstrated that *Nod2* knockdown preserved ECM content in IL-1β–treated cultures compared with control siRNA-treated groups (Figure 2, D and E). Given that IL-1β–induced chondrocyte apoptosis also contributes to ECM loss, we next examined whether *Nod2* knockdown could mitigate apoptosis. Indeed, *Nod2* knockdown significantly reduced IL-1β–induced chondrocyte apoptosis (10.66% ± 1.01% versus 19.99% ± 1.50%, *P* < 0.0001) (Figure 2F). Based on these results, we next sought to explore the in vivo role of NOD2 in OA progression using mouse models.

### Adenovirus-mediated Nod2 knockdown attenuates cartilage degradation in mouse OA joint.

**Figure 3:**
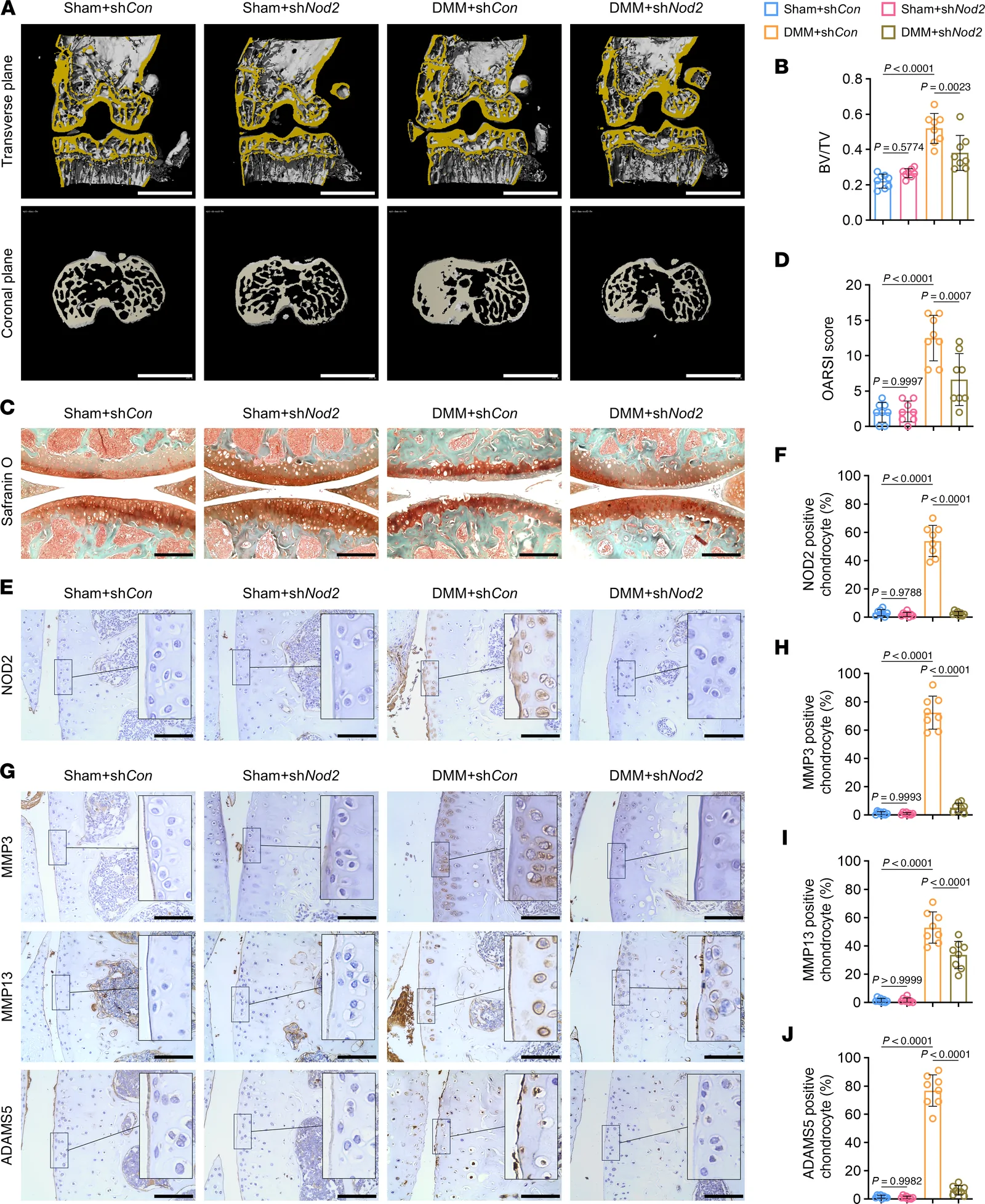
Adenovirus-mediated *Nod2* knockdown attenuates cartilage degradation in mouse OA joints. (**A**) Reconstructed μCT images of mouse knee joints following *Nod2* knockdown and DMM surgery. Yellow overlays indicate the sectional plane in the 3D reconstruction. Scale bar: 1 mm. (**B**) Quantification of bone volume/total volume (BV/TV) in tibial subchondral bone. *n* = 8 mice/group. One-way ANOVA with Tukey’s multiple-comparison test. (**C**) Safranin O/Fast Green staining of knee joint sections. Scale bar: 100 μm. (**D**) OARSI scores for cartilage degradation based on Safranin O/Fast Green staining. *n* = 8 mice/group. One-way ANOVA with Tukey’s multiple-comparison test. (**E**) IHC staining of knee sections for NOD2. Scale bar: 50 μm. (**F**) Quantification of positively stained chondrocytes for NOD2. *n* = 8 mice/group. One-way ANOVA with Tukey’s multiple-comparison test. (**G**) IHC staining of knee sections for MMP3, MMP13, and ADAMTS5. Scale bar: 50 μm. (**H**–**J**) Quantification of positively stained chondrocytes for MMP3, MMP13, and ADAMTS5. *n* = 8 mice/group. One-way ANOVA with Tukey’s multiple-comparison test. Data are presented as mean ± SD.

To investigate the role of NOD2 in vivo, we generated adenoviruses expressing shRNA targeting *Nod2* for intraarticular injection into mouse knee joints. One week following adenoviral delivery, DMM surgery was performed to induce OA (20). As subchondral bone sclerosis and osteophyte formation are hallmarks of OA progression (23), we used μCT and 3D reconstruction to evaluate structural changes in the joint. Compared with sham operated controls, DMM surgery led to densification of the tibial subchondral bone plate and the development of discernible osteophytes at the joint margins (Figure 3, A and B). The increased bone volume per total volume (BV/TV) further confirmed the occurrence of subchondral bone sclerosis in the DMM group. Notably, *Nod2* knockdown reversed these pathological changes, resulting in both lower BV/TV ratios and qualitatively reduced osteophyte formation (BV/TV: 0.38 ± 0.10 versus 0.52 ± 0.08, *P* = 0.0023). These findings suggest that *Nod2* deficiency exerts a protective effect on subchondral bone remodeling during OA progression. Histological analysis using Safranin O/Fast Green staining revealed substantial cartilage degradation at 8 weeks after DMM surgery, whereas *Nod2* knockdown significantly preserved cartilage integrity (Figure 3C). Quantification using the OARSI scoring system demonstrated a significant reduction in cartilage damage in the sh*Nod2* group compared with the sh*Con* group after surgery (6.63 ± 3.66 versus 12.50 ± 3.21, *P* = 0.0007) (24) (Figure 3D). IHC staining confirmed efficient *Nod2* knockdown in joint tissues (Figure 3, E and F). Furthermore, IHC revealed that catabolic enzymes elevated by DMM surgery, such as MMP3, MMP13, and ADAMTS5, were markedly reduced in the *Nod2* knockdown group (Figure 3, G–J). Although NOD2 levels were increased in the synovium following DMM surgery, and intraarticular injection of adenoviruses expressing *Nod2*-shRNA successfully suppressed its expression, synovial inflammation was not markedly altered. Specifically, the synovitis score, as assessed by H&E staining (25), as well as the DMM-induced infiltration of CD45^+^ leukocytes and the expression of the inflammatory mediator IL-1β, showed no significant differences between the control and knockdown groups (Supplemental Figure 1). Together, these findings indicate that intraarticular *Nod2* knockdown by adenoviruses attenuates cartilage degradation and subchondral bone remodeling in OA, without significantly altering synovial inflammation.

### cKO of Nod2 in chondrocytes prevents OA development.

**Figure 4:**
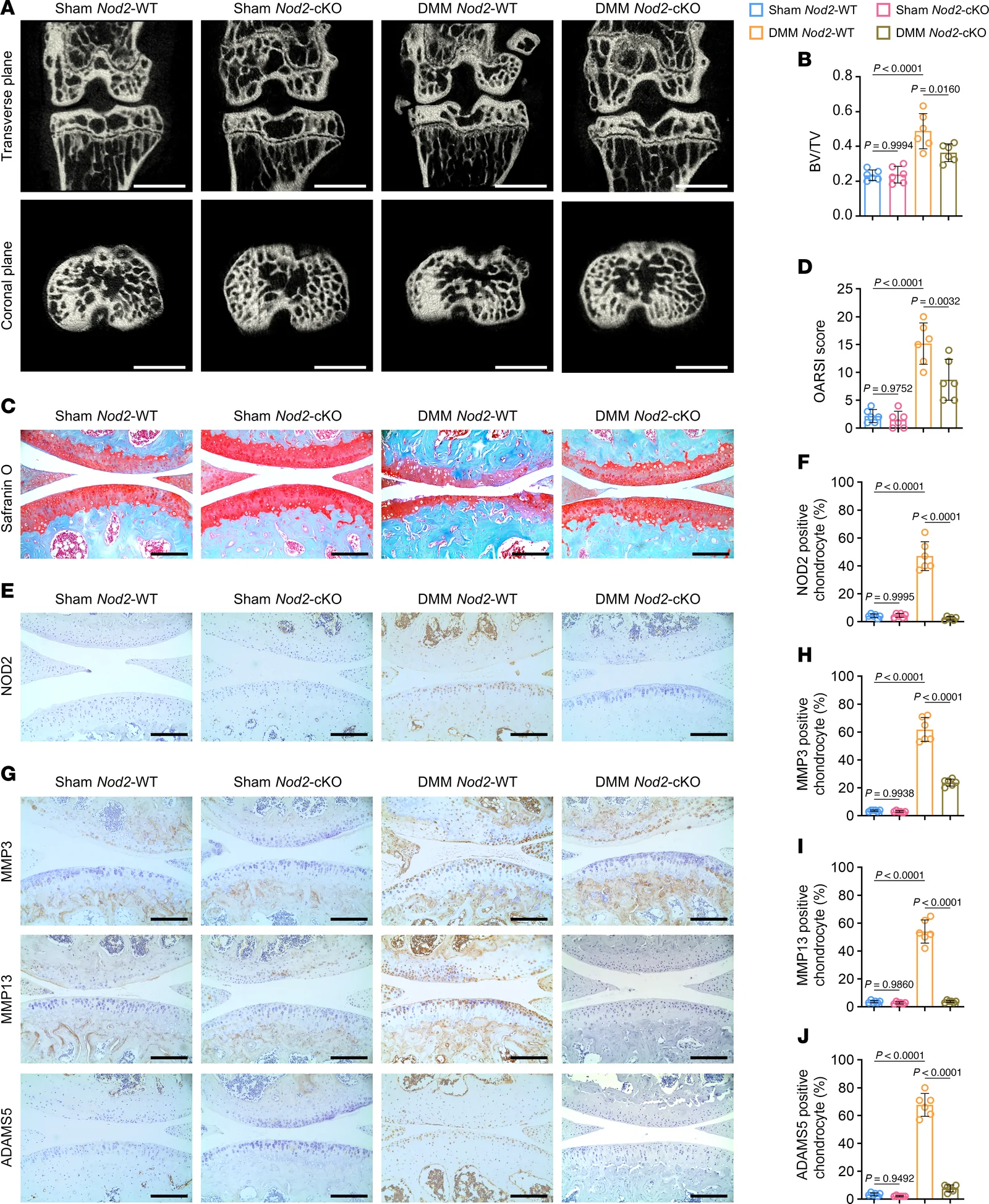
Conditional KO of *Nod2* in chondrocytes prevents OA development. (**A**) Reconstructed μCT images of mouse knee joints following *Nod2* KO and DMM surgery. Scale bar: 1 mm. (**B**) Quantification of bone volume/total volume (BV/TV) in tibial subchondral bone. *n* = 6 mice/group. One-way ANOVA with Tukey’s multiple-comparison test. (**C**) Safranin O/Fast Green staining of knee joint sections. Scale bar: 100 μm. (**D**) OARSI scores for cartilage degradation based on Safranin O/Fast Green staining. *n* = 6 mice/group. One-way ANOVA with Tukey’s multiple-comparison test. (**E**) IHC staining of knee sections for NOD2. Scale bar: 100 μm. (**F**) Quantification of positively stained chondrocytes for NOD2. *n* = 6 mice/group. One-way ANOVA with Tukey’s multiple-comparison test. (**G**) IHC staining of knee sections for MMP3, MMP13, and ADAMTS5. Scale bar: 100 μm. (**H**–**J**) Quantification of positively stained chondrocytes for MMP3, MMP13, and ADAMTS5. *n* = 6 mice/group. One-way ANOVA with Tukey’s multiple-comparison test. Data are presented as mean ± SD.

**Figure 5:**
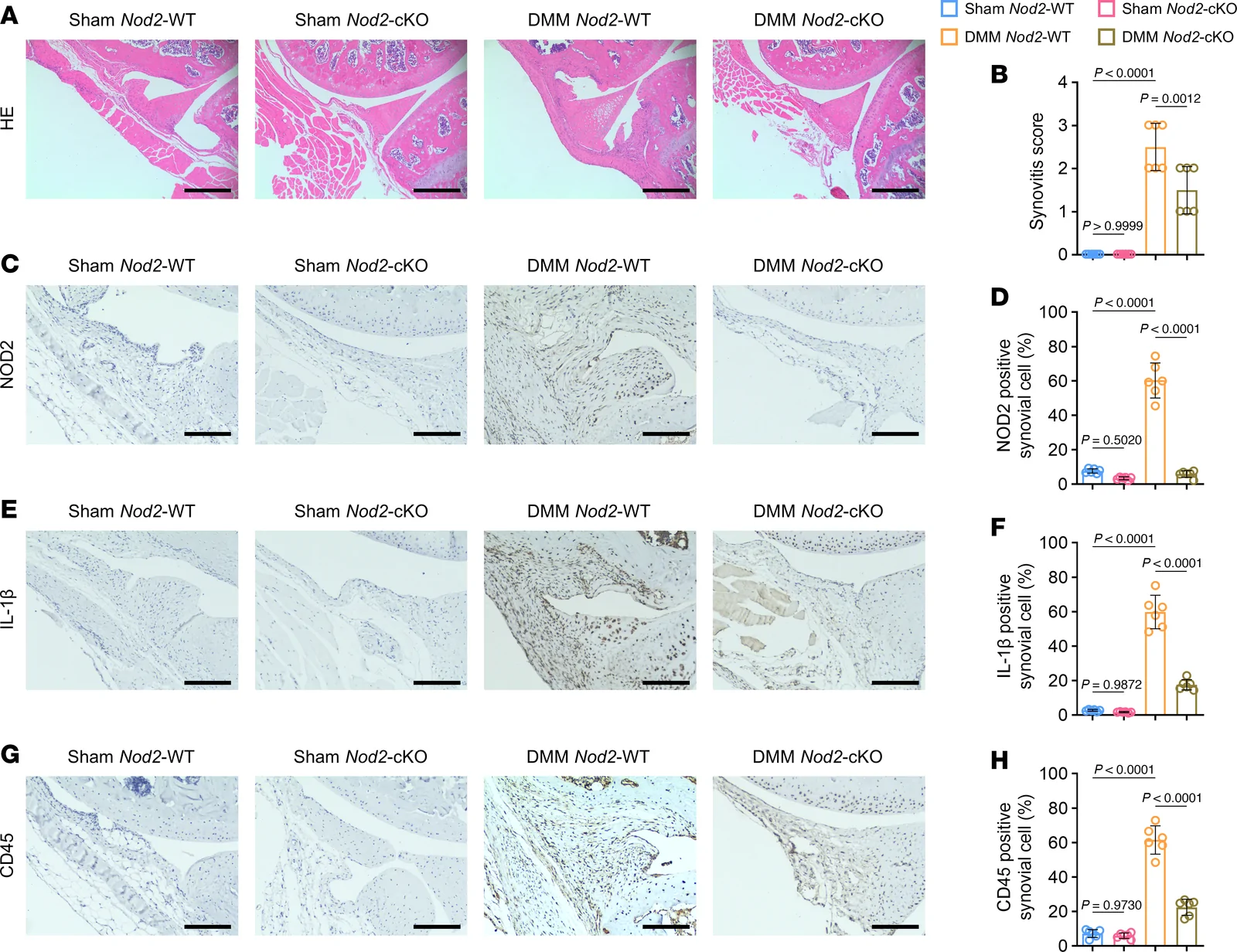
Conditional KO of *Nod2* in chondrocytes attenuates synovial inflammation. (**A**) H&E staining of mouse knee joints following *Nod2* knockout and DMM surgery. Scale bar: 200 μm. (**B**) Quantification of synovitis grading scores based on H&E staining. *n* = 6 mice/group. One-way ANOVA with Tukey’s multiple-comparison test. (**C**) IHC staining of knee sections for NOD2. Scale bar: 100 μm. (**D**) Quantification of positively stained synoviocytes for NOD2. *n* = 6 mice/group. One-way ANOVA with Tukey’s multiple-comparison test. (**E**) IHC staining of knee sections for IL-1β. Scale bar: 100 μm. (**F**) Quantification of positively stained synoviocytes for IL-1β. *n* = 6 mice/group. One-way ANOVA with Tukey’s multiple-comparison test. (**G**) IHC staining of knee sections for CD45. Scale bar: 100 μm. (**H**) Quantification of positively stained synoviocytes for CD45. *n* = 6 mice/group. One-way ANOVA with Tukey’s multiple-comparison test. Data are presented as mean ± SD.

Given the elevated expression of NOD2 in both osteoarthritic synovium and cartilage, and the high immunogenicity of adenoviruses, which may incite nonspecific synovial inflammation, we further investigated its functional role using a chondrocyte-specific cKO model. *Nod2*^fl/fl^ mice were crossed with *Col2a1*-CreER mice to generate chondrocyte-specific *Nod2*-cKO. Both *Nod2*-cKO and WT littermates (*Nod2*-WT) were treated with tamoxifen at 3 weeks of age to achieve optimal *Nod2* KO in articular cartilage (26), followed by DMM surgeries at 12 weeks of age to induce OA. At 8 weeks after surgery, *Nod2*-WT mice displayed characteristic OA pathology, including tibial subchondral bone densification (Figure 4, A and B) and significant cartilage degradation (Figure 4, C and D), accompanied by increased expression of catabolic enzymes such as MMP3, MMP13, and ADAMTS5 (Figure 4, E–J). In contrast, *Nod2*-cKO mice were protected from these pathological changes, maintaining subchondral bone structure (BV/TV: 0.36 ± 0.05 versus 0.48 ± 0.10, *P* = 0.0160) and cartilage integrity (OARSI score: 8.67 ± 3.67 versus 15.17 ± 3.71, *P* = 0.0032). IHC staining confirmed efficient NOD2 deletion in chondrocytes and revealed a corresponding reduction in catabolic enzyme expression in *Nod2*-cKO mice. We further examined the synovial pathology in *Nod2*-cKO mice. This was prompted by the observation that adenovirus-mediated *Nod2* knockdown did not significantly alter DMM-induced synovitis, a result that may have been confounded by the inherent immunogenicity typically associated with viral vectors. Surprisingly, the specific ablation of *Nod2* in chondrocytes resulted in a significant reduction in synovial inflammation compared with control *Nod2*-WT mice. Specifically, *Nod2*-cKO mice exhibited attenuated synovial hyperplasia and decreased infiltration of inflammatory cells (synovitis score: 1.50 ± 0.55 versus 2.50 ± 0.55, *P* = 0.0012) (Figure 5, A–H). These findings suggest that targeted ablation of *Nod2* in chondrocytes was associated with a reduction in synovial inflammation. This protective effect potentially results from decreased release of cartilage-derived DAMPs into the joint space, thereby attenuating the catabolic crosstalk between cartilage and synovium that drives OA progression.

### Activation of NOD2 by MDP is not sufficient to promote cartilage degradation.

**Figure 6:**
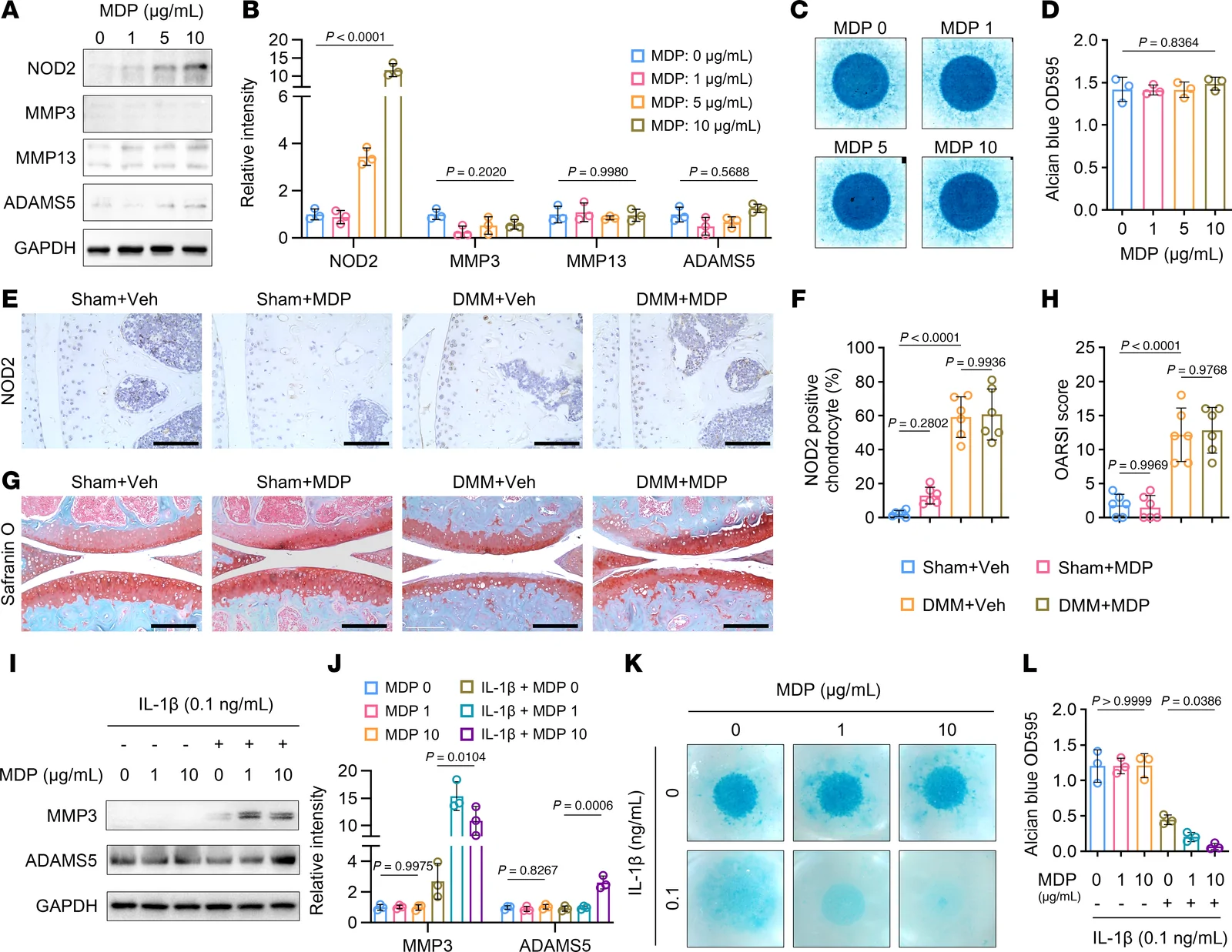
Activation of NOD2 by MDP alone is insufficient to drive cartilage degradation. (**A** and **B**) Western blot analysis and densitometric quantification of NOD2, MMP3, MMP13, and ADAMTS5 protein levels in primary mouse chondrocytes treated with MDP (0–10 μg/mL) for 72 h. *n* = 3 independent experiments. One-way ANOVA with Tukey’s multiple-comparison test. (**C**) Alcian blue staining of micromass cultures of primary mouse chondrocytes treated with MDP (0–10 μg/mL) for 2 weeks. (**D**) Quantification of proteoglycan content by absorbance of dissolved Alcian blue at 595 nm. *n* = 3 independent experiments. One-way ANOVA with Tukey’s multiple-comparison test. (**E**) IHC staining of knee sections for NOD2. Scale bar: 50 μm. (**F**) Quantification of positively stained chondrocytes for NOD2. *n* = 6 mice/group. One-way ANOVA with Tukey’s multiple-comparison test. (**G**) Safranin O/Fast Green staining of knee joint sections. Scale bar: 100 μm. (**H**) OARSI scores for cartilage degradation based on Safranin O/Fast Green staining. *n* = 6 mice/group. One-way ANOVA with Tukey’s multiple-comparison test. (**I** and **J**) Western blot analysis and densitometric quantification of MMP3 and ADAMTS5 protein levels following indicated concentrations of MDP and IL-1β treatment (0.1 ng/mL for 72 h). *n* = 3 independent experiments. One-way ANOVA with Tukey’s multiple-comparison test. (**K**) Alcian blue staining of micromass cultures of primary mouse chondrocytes treated with indicated concentrations of MDP and IL-1β treatment (0.1 ng/mL) for 2 weeks. (**L**) Quantification of proteoglycan content by absorbance of dissolved Alcian blue at 595 nm. *n* = 3 independent experiments. One-way ANOVA with Tukey’s multiple-comparison test. Data are presented as mean ± SD.

As we have observed earlier, MDP alone can activate NOD2 in chondrocytes (Figure 1O); we investigated whether MDP-mediated NOD2 activation is sufficient to drive cartilage degradation. As expected, MDP treatment significantly increased NOD2 protein levels in primary mouse chondrocytes. However, MDP stimulation did not efficiently induce the expression of catabolic enzymes, including MMP3, MMP13, or ADAMTS5 (Figure 6, A and B). In high-density chondrocyte micromass cultures, MDP treatment failed to promote ECM degradation, as shown by Alcian blue staining (Figure 6, C and D). Furthermore, intra-articular injection of MDP following DMM surgery in the mouse OA model did not exacerbate cartilage damage. Although MDP increased NOD2 expression in sham-operated joints, its effect was minimal compared with the robust NOD2 activation induced by DMM surgery, as indicated by IHC staining (Figure 6, E and F). Safranin O/Fast Green staining and OARSI scoring revealed no significant differences in cartilage integrity between MDP- and vehicle-treated groups (OARSI score: 12.83 ± 3.37 versus 12.17 ± 3.92, *P* = 0.9768) (Figure 6, G and H). To further investigate whether NOD2 requires a proinflammatory context to exert its pathological effects, we performed costimulation experiments using low-dose IL-1β (0.1 ng/mL) and MDP in primary mouse chondrocytes. While MDP alone had minimal effects, the combination of IL-1β and MDP led to a synergistic increase in the expression of catabolic enzymes, including MMP3 and ADAMTS5, compared with IL-1β treatment alone (Figure 6, I and J). Consistent with these molecular changes, Alcian blue staining demonstrated a more pronounced loss of proteoglycan content in the costimulated group (Figure 6, K and L). These results indicate that MDP-mediated NOD2 activation alone is insufficient to trigger cartilage degradation and NOD2 functions as an inflammatory amplifier, thereby driving cartilage matrix degradation through a synergistic mechanism.

### NOD2 amplifies inflammatory signals by regulating TRAF6 stability and ubiquitination.

**Figure 7:**
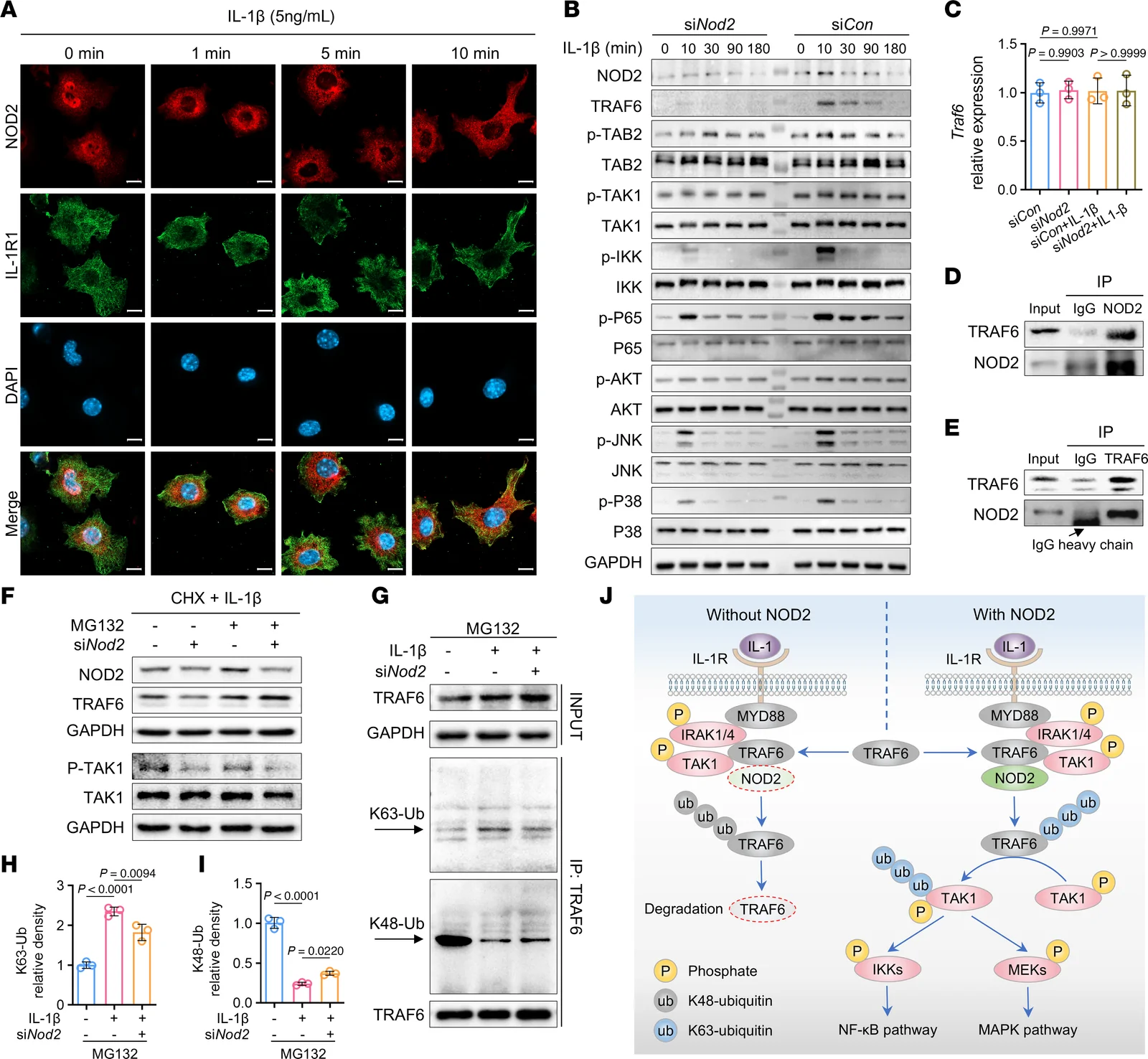
NOD2 amplifies inflammatory signals by regulating TRAF6 stability and ubiquitination. (**A**) Immunofluorescence images showing the subcellular localization of NOD2 and IL-1R1 in primary mouse chondrocytes stimulated with IL-1β (5 ng/mL) over the indicated time course. Scale bar: 10 μm. (**B**) Western blot analysis of NOD2 and IL-1β signaling pathway activation (TRAF6, p-TAB2, p-TAK1, p-IKK, p-p65, p-AKT, p-JNK, and p-p38) in primary mouse chondrocytes following *Nod2* knockdown and IL-1β treatment (5 ng/mL) for the indicated durations. (**C**) qPCR analysis of *Traf6* mRNA levels in primary mouse chondrocytes. *n* = 3 independent experiments. One-way ANOVA with Tukey’s multiple-comparison test. (**D** and **E**) Coimmunoprecipitation (Co-IP) and Western blot analysis of endogenous and exogenous interactions between TRAF6 and NOD2. (**F**) Western blot analysis of NOD2 and TRAF6 protein levels in chondrocytes treated with cycloheximide (CHX) and IL-1β (5 ng/mL), with or without *Nod2* knockdown and MG132. (**G**) IP and Western blot analysis of TRAF6 ubiquitination status. (**H** and **I**) Densitometric quantification of K63-linked and K48-linked ubiquitination of TRAF6. *n* = 3 independent experiments. One-way ANOVA with Tukey’s multiple-comparison test. (**J**) Schematic illustration showing that NOD2 stabilizes TRAF6 and amplifies IL-1β signaling by modulating K63- and K48-linked ubiquitination. Data are presented as mean ± SD.

As an intracellular pattern recognition receptor, NOD2 is typically localized in the cytoplasm under basal conditions. However, its recruitment to membrane compartments is essential for initiating downstream inflammatory responses (27). In primary mouse chondrocytes treated with IL-1β, we observed rapid translocation of NOD2 to the plasma membrane, where it colocalized with the type I IL-1 receptor (IL-1R1), suggesting a potential role for NOD2 in initiating the IL-1β signaling cascade (Figure 7A). IL-1β signaling in chondrocytes involves the activation of NF-κB and MAPK pathways through shared kinase cascades involving tumor necrosis factor receptor–associated factor 6 (TRAF6), TGF-β–activated kinase 1 binding protein 1 (TAB1), and TGF-β–activated kinase 1 (TAK1) (28). To elucidate the role of NOD2 in this pathway, we analyzed key components downstream of IL-1β stimulation. Knockdown of *Nod2* in primary chondrocytes led to reduced TRAF6 protein levels and decreased phosphorylation of TAB1 and TAK1. Consistently, activation of both NF-κB and MAPK pathways was suppressed, as shown by reduced phosphorylation of IKKs, P65, JNK, and P38 at 10 minutes after IL-1β treatment (Figure 7B). Since NOD2 protein levels did not markedly increase until 72 hours after IL-1β treatment (Figure 1, I and J), we hypothesized that the basal level of NOD2 is sufficient to mediate the early cellular response to IL-1β stimulation.

TRAF6 is a central adaptor protein involved in transducing signals from multiple inflammatory cytokines, including IL-1β and TNF-α, and functions upstream of several kinase cascades (29). We next examined whether NOD2 regulates IL-1β signaling by modulating TRAF6 activity. qPCR analysis confirmed that neither IL-1β stimulation nor *Nod2* knockdown altered *Traf6* mRNA expression (Figure 7C), suggesting posttranscriptional regulation. At the protein level, coimmunoprecipitation of both endogenous and exogenous proteins revealed that NOD2 interacts with TRAF6 upon IL-1β treatment (Figure 7, D and E). The activity and stability of TRAF6 are regulated by distinct ubiquitin linkages; K63-linked ubiquitination promotes signal transduction, while K48-linked ubiquitination targets TRAF6 for proteasomal degradation (30). To determine whether NOD2 influences TRAF6 stability, we treated cells with cycloheximide (CHX) to block new protein synthesis and MG132 to inhibit proteasomal degradation. *Nod2* knockdown reduced IL-1β–induced TRAF6 protein levels, whereas this effect was negated in the presence of MG132, suggesting that NOD2 protects TRAF6 from proteasome-dependent degradation (Figure 7F). Notably, MG132-mediated restoration of TRAF6 protein levels failed to rescue downstream TAK1 activation, highlighting the critical role of proper posttranslational modification in enabling TRAF6 signaling. We next assessed the ubiquitination status of TRAF6. Following IL-1β stimulation in the presence of MG132, we immunoprecipitated TRAF6 and examined K63- and K48-linked ubiquitination. IL-1β increased K63-linked and decreased K48-linked ubiquitination of TRAF6, whereas *Nod2* knockdown inhibited K63-linked ubiquitination (relative level: 1.83 ± 0.21 versus 2.35 ± 0.11, *P* = 0.0094) and enhanced K48-linked ubiquitination (relative level: 0.37 ± 0.03 versus 0.24 ± 0.02, *P* = 0.0220) of TRAF6 (Figure 7, G–I). Together, these findings demonstrate that NOD2 binds to TRAF6 upon IL-1β stimulation and promotes its activation by enhancing K63-linked ubiquitination while suppressing K48-linked degradation signals. This dual regulatory mechanism stabilizes TRAF6 and amplifies IL-1β induced inflammatory signaling in chondrocytes (Figure 7J).

### NOD2 is a potential therapeutic target for OA.

**Figure 8:**
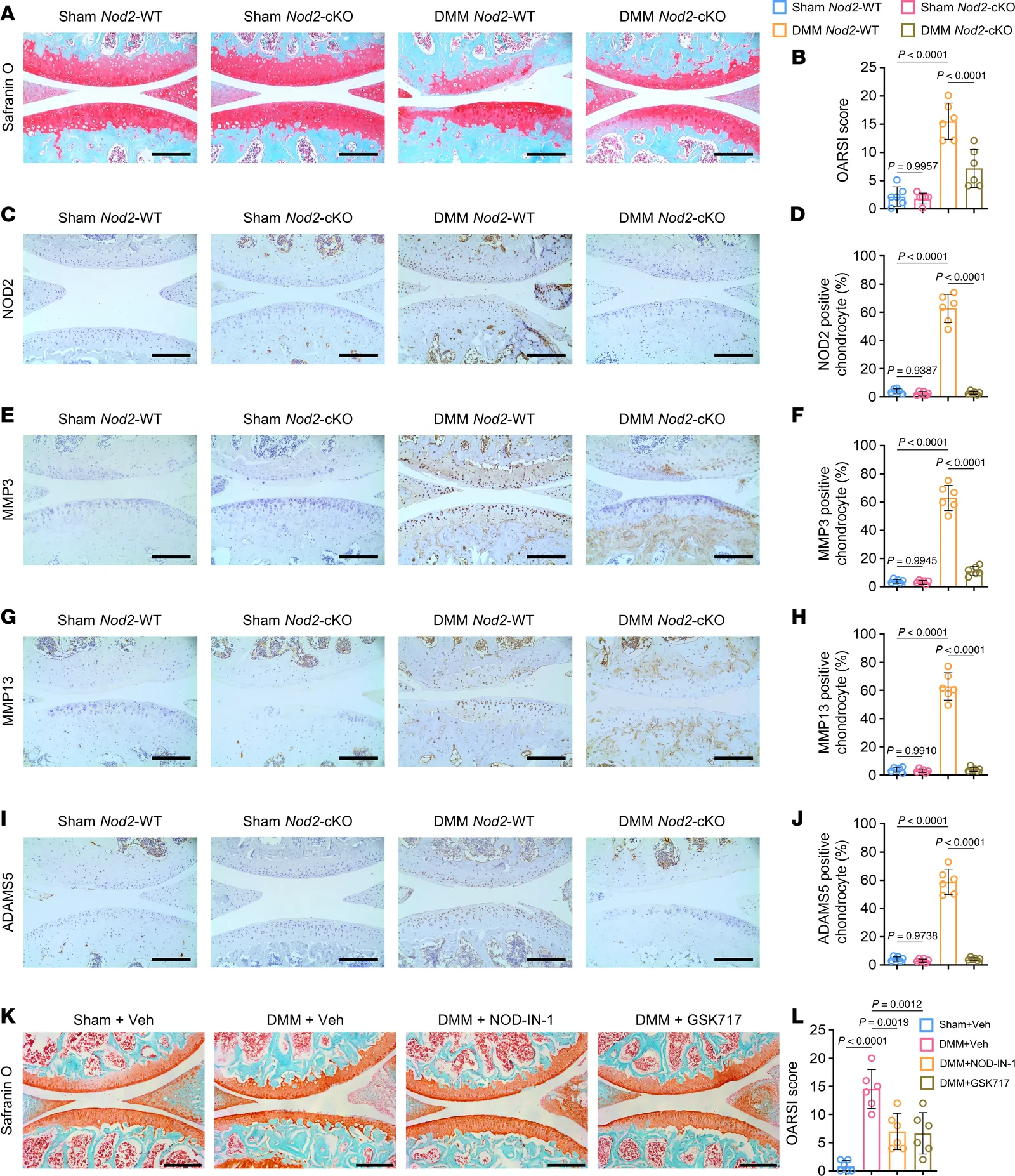
Post-DMM interventions targeting NOD2 attenuate OA development. (**A**) Safranin O/Fast Green staining of knee joint sections following DMM surgery and *Nod2* KO. Scale bar: 100 μm. (**B**) OARSI scores for cartilage degradation based on Safranin O/Fast Green staining. *n* = 6 mice/group. One-way ANOVA with Tukey’s multiple-comparison test. (**C**) IHC staining of knee sections for NOD2. Scale bar: 100 μm. (**D**) Quantification of positively stained chondrocytes for NOD2. *n* = 6 mice/group. One-way ANOVA with Tukey’s multiple-comparison test. (**E**–**J**) IHC staining of knee sections and quantification of positively stained chondrocytes for MMP3, MMP13, and ADAMTS5. Scale bar: 100 μm. *n* = 6 mice/group. One-way ANOVA with Tukey’s multiple-comparison test. (**K**) Safranin O/Fast Green staining of knee joint sections following DMM surgery and NOD2 antagonists. Scale bar: 100 μm. (**L**) OARSI scores for cartilage degradation based on Safranin O/Fast Green staining. *n* = 6 mice/group. One-way ANOVA with Tukey’s multiple-comparison test. Data are presented as mean ± SD.

To rigorously assess the therapeutic potential of targeting NOD2 in established OA, we evaluated the effects of post-DMM intervention using 3 complementary approaches: adenovirus-mediated knockdown, inducible chondrocyte-specific knockout, and pharmacological inhibition with small-molecule antagonists. The mouse OA model was induced via DMM surgery, and all therapeutic interventions were initiated 1 week after DMM, a time point reflecting early-stage OA pathology. At 8 weeks after DMM, adenovirus-mediated *Nod2* knockdown significantly attenuated further cartilage structural deterioration and suppressed the expression of key catabolic enzymes, including MMP3, MMP13, and ADAMTS5 (Supplemental Figure 2). Similarly, the specific KO of *Nod2* in chondrocytes post-DMM yielded robust chondroprotective effects, characterized by preserved cartilage morphology (OARSI score: 7.17 ± 3.37 versus 15.50 ± 3.21, *P* < 0.0001) and reduced catabolic enzymes (Figure 8, A–J). Two independent inhibitors were tested: NOD-IN-1, a nonselective inhibitor targeting both NOD1 and NOD2, and GSK717, a selective NOD2 inhibitor (31, 32). The mouse OA model was induced via DMM surgery, and inhibitors were intraarticularly injected twice a week for 7 weeks after surgery. Histological analysis of knee sections using Safranin O/Fast Green staining revealed that both NOD-IN-1 and GSK717 significantly attenuated cartilage degradation compared with vehicle-treated controls. This was evidenced by increased cartilage thickness, reduced cartilage damage, and lower OARSI histological scores (7.00 ± 3.23 versus 14.50 ± 3.45, *P* = 0.0019 for NOD-IN-1 versus vehicle; 6.67 ± 3.67 versus 14.50 ± 3.45, *P* = 0.0012 for GSK717 versus vehicle) (Figure 8, K and L). These findings support NOD2 as a promising therapeutic target for the prevention and treatment of OA.

## Discussion

Despite substantial advances in understanding OA pathogenesis, effective disease-modifying treatments remain elusive (33). Nonsteroidal antiinflammatory drugs (NSAIDs) are currently recommended as the first-line pharmacologic option to alleviate symptoms, as endorsed by several major clinical guidelines (34–36). However, multiple phase II and III clinical trials targeting inflammation, particularly those employing monoclonal antibodies or small-molecule inhibitors against TNF-α, IL-1β, and IL-6, have shown limited efficacy in halting disease progression (33). These findings suggest that inhibition of individual cytokines may be insufficient, given the complexity and redundancy of inflammatory signaling in OA. Since inflammatory cytokines act in parallel to promote cartilage destruction by inducing a broad range of matrix-degrading enzymes, an alternative strategy may lie in targeting converging intracellular signaling hubs. In this study, we investigated the role of NOD2, an intracellular innate immune sensor, as a central amplifier of inflammation in OA. Unlike existing therapies that target extracellular cytokines or matrix metalloproteinases, our findings highlight NOD2 as a mediator of chondrocyte-intrinsic inflammatory responses and a viable target for therapeutic intervention. Both genetic and pharmacologic inhibition of NOD2 reduced cartilage degradation and subchondral bone remodeling in mouse OA models, supporting the potential for NOD2-targeted strategies in OA treatment.

NOD2 is best known as a cytosolic pattern recognition receptor expressed in antigen-presenting cells (APCs), such as macrophages and dendritic cells, where it responds to bacterial peptidoglycan-derived MDP and endogenous danger-associated molecular patterns (DAMPs) (37). Its involvement in autoimmune arthritis, including Lyme arthritis and pediatric granulomatous arthritis, is supported by both clinical associations and experimental validations (38, 39). In the context of OA, however, the role of NOD2 has been variably reported, with studies showing contradictory findings (17, 18). While elevated NOD2 expression has been observed in OA synovial tissue, intraarticular injection of NOD2-overexpressing lentivirus alleviated pathological changes in a collagenase-induced mouse OA model (17). Additionally, a genomic analysis of 150 independent families identified rare alleles in NOD-RIPK2 pathway genes associated with multiple types of familial OA (18). Despite these insights, the cell type–specific function of NOD2, particularly in chondrocytes, has not been well characterized. Using chondrocyte-specific KO mice, our study provides direct evidence that NOD2 acts as a key intracellular amplifier of inflammatory signaling in chondrocytes and contributes to cartilage degradation in OA.

This study primarily focused on the role of NOD2 in cartilage degradation and its underlying molecular mechanisms. However, several important aspects remain to be explored. For instance, the specific triggers of NOD2 activation in osteoarthritic cartilage are not yet fully elucidated. While joint wear and tear or injury may release various molecules into the synovial fluid that act as DAMPs (40), the systemic translocation of bacterial products represents another possible source of NOD2 ligands (41). Growing evidence suggests that increased intestinal permeability in patients with OA may allow gut microbiota derived peptidoglycans to enter the systemic circulation and subsequently infiltrate joint tissues (42). Identifying whether NOD2 activation in chondrocytes is primarily driven by gut-derived bacterial fragments or by specific endogenous DAMPs warrants further investigation. Moreover, this study did not directly compare the roles of NOD2 in cartilage and synovium. While cartilage is composed exclusively of chondrocytes, the synovium contains fibroblast-like synoviocytes and macrophage-like synovial cells in the intimal layer, along with a more heterogeneous cellular composition in the subintimal region (43). As central components of the joint’s innate immune system, synovial macrophages help maintain homeostasis by clearing cellular debris and inflammatory stimuli (44). The elevated NOD2 expression observed in OA synovium suggests that it may play a broader role in shaping the joint’s inflammatory microenvironment (17). Conditional deletion of *Nod2* in macrophage lineages could help clarify its contribution to synovial inflammation and OA pathogenesis.

In summary, this study demonstrated that NOD2 is activated in osteoarthritic cartilage and contributes to cartilage degradation by regulating TRAF6 activity. Both pharmacological inhibition and chondrocyte-specific KO of *Nod2* effectively attenuated OA progression in mouse models. These findings provide insights into the molecular mechanisms driving cartilage degradation in OA and highlight NOD2 as a promising therapeutic target.

## Methods

### Sex as a biological variable.

Human cartilage samples were collected from both male and female patients. All animal experiments were conducted using male mice to minimize variability related to hormonal cycling in females and to maintain consistency with established protocols in DMM surgery-induced OA models.

### Human cartilage samples.

Osteoarthritic cartilage specimens were obtained from patients undergoing total knee arthroplasty for clinically and radiographically diagnosed advanced OA (Kellgren-Lawrence grade 3 or 4) (45). Healthy control cartilage was collected from donors undergoing lower limb amputation due to trauma or tumor, who had no prior history or clinical evidence of OA (Kellgren-Lawrence grade 0). To ensure anatomical consistency, all cartilage was harvested from the weight-bearing region of the medial tibial plateau. For the OA group, full-thickness cartilage was specifically collected from macroscopically preserved regions adjacent to the lesion site (OARSI grade 3.2 ± 0.3, 3–3.5) (46) to ensure sufficient tissue integrity and cellularity for molecular analysis. Demographic characteristics, including age, sex, and BMI, were balanced between the 2 groups, as summarized in Supplemental Table 1.

### Animal models of OA.

Animals were supplied by the University Laboratory Animal Center and raised in specific pathogen-free conditions with free access to food and water. DMM was used to produce OA models following previously published protocols (19). For the *Nod2*-knockdown experiment, mice (C57BL/6, 12 weeks old, male) were randomly divided into 4 groups and pretreated with adenovirus (1.5 × 10^8^ PFU in 10 μL) carrying shRNA targeting *Nod2* or a control sequence by intraarticular injection one week before the surgery. These mice received another intraarticular injection of the above adenovirus at 4 weeks after surgery and were sacrificed at 8 weeks after surgery. For the therapeutic experiment, adenovirus (1.5 × 10^8^ PFU in 10 μL) carrying shRNA targeting *Nod2* or a control sequence were intraarticular injected 1 week after DMM surgery. For the MDP treatment experiment, mice (C57BL/6, 12 weeks old, male) were randomly divided into 4 groups. After 1 week of recovery from DMM or sham surgery, animals received intraarticular injections of MDP (5 μg in 10 μL saline) or vehicle twice a week for 7 weeks before sacrifice. For the NOD2 inhibitor injection experiment, mice (C57BL/6, 12 weeks old, male) were randomly divided into 4 groups. After 1 week of recovery from DMM or sham surgery, animals received intraarticular injections of NOD-IN-1 (1 mM, dissolved in 2% DMSO plus 98% saline), GSK717 (1 mM, dissolved in 2% DMSO plus 98% saline), or vehicle twice a week for 7 weeks before sacrifice. This frequency is effective for maintaining sufficient drug concentrations within the joint space while minimizing the adverse effects associated with excessive joint punctures as demonstrated by previous studies (47, 48).

### Nod2-cKO mice.

The *Nod2*^fl/fl^ mice (C57BL/6JGpt-Nod2^em1Cflox^/Gpt, strain no. T005791) and *Col2a1*-CreER mice (C57BL/6JGpt-Col2a1^em1Cin(P2A-CreERT2)^/Gpt, strain no. T066454) were generated by GemPharmatech Co. Ltd. *Nod2*^fl/fl^ mice were crossed with *Col2a1*-CreER mice to generate *Nod2* flox/*Col2a1*-CreER (*Nod2*-cKO) mice. While the *Col2a1*-CreER (*Nod2*-WT) mice were used as wild-type controls. Both *Nod2*-cKO and *Nod2*-WT mice were treated with tamoxifen (6 mg per day for 5 days) by oral gavage at 3 weeks of age to achieve optimal *Nod2* knockout in articular cartilage (25). The DMM surgeries were performed at 12 weeks of age. For the therapeutic experiment, both *Nod2*-cKO and *Nod2*-WT mice were treated with tamoxifen (6 mg per day for 5 days) by oral gavage adenovirus starting one week post DMM surgery. All mice were sacrificed at 8 weeks after surgery.

### μCT imaging.

Knee joints were fixed in 4% buffered formaldehyde for 2 days before being scanned with a vivaCT40 μCT instrument (Scanco Medical). Scans were obtained at 100 kV and 98 μA, with the resolution set to 10.5 μm. The built-in software was used to reconstruct and analyze images. The bone volume per total volume (BV/TV) was determined for statistical analysis.

### Histological staining and analysis.

Knee joints were decalcified for 4 weeks using 10% EDTA solution and then processed for embedding in paraffin wax. The tissues were cut into 4 μm–thick sagittal sections. Safranin O/Fast Green staining was performed following standard protocol. IHC for NOD2, MMP3, MMP13, and ADAMTS5 was performed using a DAB Histostain-SP Kit. Histological measurements and images were observed using an Eclipse Ti-S inverted microscope (Nikon Instruments). Cartilage degradation in the DMM model was assessed using the Osteoarthritis Research Society International (OARSI) scoring system on Safranin O–stained sagittal sections. For each joint, 4 anatomical quadrants (anterior and posterior of the femoral and tibial medial compartments) were graded on a scale of 0–6. The severity of OA was reported as the sum of the scores from these 4 quadrants (total score ranging from 0 to 24), as recommended by the OARSI histopathology initiative (23). A synovitis grading system was used to assess the severity of synovitis (24).

### Chondrocyte and micromass culture.

Human primary chondrocytes were obtained from healthy cartilage. Mouse chondrocytes were collected from the knees of 3-day-old C57BL/6 mice. Briefly, articular cartilage was dissected and cut into pieces before being digested in trypsin at 37°C for 30 minutes. The tissue suspension was then centrifuged and the supernatant was discarded. The remaining tissue was digested in 0.2% type II collagenase at 37°C until no cartilage particles remained. The chondrocytes were then cultured in DMEM/F12 at 37°C with 5% CO_2_ supplied. After 24 hours, floating cells were discarded, and the adherent cells were continuously cultured as chondrocytes (49, 50). For micromass culture, 5 × 10^5^ chondrocytes in 20 μL were seeded in the center of 24-well plates for 2 hours before the addition of 1 mL of DMEM/F12 medium. Micromasses were cultured for 2 weeks before staining.

### Adenovirus and siRNA.

Adenovirus carrying shRNA targeting mouse *Nod2* (5′-GCGAGCACUUCCAUUCCAU-3′) and control sequence were prepared and concentrated to 5 × 10^11^ PFU/mL by HanBio Technology. For the animal experiment, adenoviruses (1.5 × 10^8^ PFU in 10 μL) were injected intraarticularly. For in vitro experiments, the chondrocytes were infected with adenovirus at a multiplicity of infection (MOI) of 30:1. siRNA targeting mouse *Nod2* (5′-GCGAGCACUUCCAUUCCAUTT-3′) was designed and synthesized by RiboBio. Transfection of siRNA was performed using Lipofectamine 3000 (Invitrogen) according to the manufacturer’s instructions.

### Apoptosis assays.

Chondrocytes were harvested, rinsed with ice-cold phosphate-buffered saline (PBS), resuspended in binding buffer (500 μL), and incubated with propidium iodide (PI; 5 μL) and annexin V–fluorescein isothiocyanate (5 μL) at 4°C in the dark for 15 minutes. The cells were then rinsed and resuspended in PBS (500 μL) and analyzed by flow cytometry (BD Accuri C6; BD Biosciences) according to the manufacturer’s instructions. Data were processed by FlowJo software (Treestar Inc.).

### RNA isolation and qPCR.

Total RNA was extracted using TRIzol reagent (Invitrogen) according to the manufacturer’s instructions. cDNA was synthesized using a RevertAid First Strand cDNA Synthesis Kit (Thermo Scientific), and qPCR was performed using a KAPA SYBR FAST qPCR Kit (Kapa Biosystems). The relative mRNA levels of target genes were calculated by the 2^–ΔΔCT^ comparative Ct method using Gapdh as a control gene and normalized to the control group (51).

### Coimmunoprecipitation and Western blotting.

The interactions of NOD2 with TRAF6 were detected in mouse chondrocytes stimulated with IL-1β using coimmunoprecipitation as previously described (52). Cell lysates were precleared by incubation with control IgG and protein A/G beads for 1 hour to block nonspecific binding to the immunoprecipitation components. For immunoprecipitation, the precleared cell lysates were incubated with the indicated antibodies and protein A/G beads overnight. The immunoprecipitates were washed 3 times with lysis buffer, boiled in SDS sample buffer, and then subjected to Western blotting using the antibodies listed below, following the manufacturer’s protocols.

### Reagents and antibodies.

Recombinant soluble human and mouse IL-1β and TNF-α were purchased from PeproTech. MDP was purchased from InvivoGen. Antibodies against NOD2 (catalog NB100-524, NBP2-27328) were obtained from Novus Biologicals; the antibodies against MMP3 (catalog sc-271230) was obtained from Santa Cruz Biotechnology; the antibodies against MMP13 (catalog ab39012) was obtained from Abcam Limited.; antibodies against ADAMTS5 (catalog BA3020) and against GAPDH (catalog BM1623) were obtained from Boster Biological Technology; the antibodies against TAB2 (catalog 14410-1-AP) was obtained from Proteintech Group; antibodies against TRAF6 (catalog 8028), p-TAB2 (catalog 8155), TAK1 (catalog 5206), p-TAK1 (catalog 4508), IKK (catalog 8943), p-IKK (catalog 2697), P65 (catalog 8242), p-P65 (catalog 3033), AKT (catalog 4691), p-AKT (catalog 4060), JNK (catalog 9252), p-JNK (catalog 4668), P38 (catalog 8690), and p-P38 (catalog 4511) were obtained from Cell Signaling Technology. Secondary antibodies were acquired from Jackson ImmunoResearch Laboratories. The basal culture medium was obtained from GE Healthcare Life Sciences.

### Statistics.

All statistical analyses were performed with GraphPad Prism 9. All in vitro experiments were performed at least 3 times independently, and the replicates for in vivo studies were indicated in the figure legends. The results are presented as means ± SD if not specifically stated. The Shapiro-Wilk test was used to test normality, and the F test was used to test equal variance. Two-tailed Student’s *t* test was used for comparisons between 2 groups, and a 1-wayANOVA followed by Dunnett’s post hoc test was used for comparisons involving more than 2 groups. Nonparametric tests were used to analyze data that failed either the normality or equal variance test. The Mann-Whitney *U* test was used for comparisons between 2 groups, and the Kruskal-Wallis test was used for comparisons among more than 2 groups. In all analyses, a *P* value less than 0.05 was considered significant. All *P* values were presented numerically.

### Study approval.

The collection and use of human cartilage samples were approved by the Ethics Committee of Tongji Hospital, Huazhong University of Science and Technology (TJ-IRB20210127), and written informed consent was obtained before sample collection. All animal experiments were approved by the IACUC of Tongji Hospital, Huazhong University of Science and Technology (TJH-202001011), and were conducted following the *Guide for the Care and Use of Laboratory Animals* (National Academies Press, 2011). All procedures involving human samples were performed in accordance with the Declaration of Helsinki.

### Data availability.

All data relevant to the study are included within the manuscript and the accompanying Supporting Data Values file.

## Author contributions

YW, S Li, FG, and LZ conceived and designed the study. YW, S Li, YD, JZ, JL, ZW, S Liang, NRM, HP, PC, and LZ performed experiments and analyzed data. YW, AC, QY, JX, and LZ provided research funds. CKFC, JX, FG, and LZ jointly supervised the study. YW, S Li, FG, and LZ wrote the manuscript with contributions from all authors. WJ provided editorial assistance, including critical review of the manuscript for clarity, organization, and presentation. He did not contribute to the study conception or design, data collection, data analysis, interpretation of the results, or the generation of the scientific content. All authors reviewed and approved the final manuscript. The order of co–first authors was determined based on YW’s primary role in initiating the project, with mutual agreement from all authors.

## Conflict of interest

The authors have declared that no conflict of interest exists.

## Funding support

- National Natural Science Foundation of China (82402820 to YW, 81902262 to LZ, 82330075 to JX, 81672168 to AC, 81601951 to QY).

## Supplementary Material

Supplemental data

Unedited blot and gel images

Supporting data values

## Version 1 08/24/2026

Electronic publication
