# Supplementary material for: Innate immune sensor NOD2 promotes cartilage degradation and osteoarthritis progression by stabilizing TRAF6 in chondrocytes
Source: JCI Insight. 2026 Aug 24;11(16):e196750. doi: 10.1172/jci.insight.196750 (PMC13502188; doi:10.1172/jci.insight.196750)
Supplement: Supplementary file 1 [file jciinsight-11-196750-s191.pdf]

Supplemental Table 1: Demographic and clinical profiles of human cartilage samples.

Characteristic	Healthy (n=3)	OA (n=3)	P-value
Age (years)	51.7 ± 12.7 (37-60)	65.7 ± 3.8 (63-70)	0.1422
Sex （male/female)	1/2	1/2	1.000
Ethnicity	East Asian (Han Chinese)	East Asian (Han Chinese)	N/A
BMI (kg/m^2)	25.9 ± 0.3	25.7 ± 1.5	0.7836
Kellgren-Lawrence Grade	0 (0-0)	3.7 ± 0.6 (3-4)	0.0004
Anatomical Location	Medial Tibial Plateau	Medial Tibial Plateau	N/A
Sampling Site	Intact	Intact (Lesion-adjacent)	N/A
OARSI Grade	0.7 ± 0.6 (0-1)	3.2 ± 0.3 (3-3.5)	0.0026

Supplemental Table 1: Demographic and clinical profiles of human cartilage samples. n = 3 donors/group. Two-tailed, unpaired Student’s t-test. Data are presented as mean ± SD.

Supplemental Figure 1. Adenovirus-mediated *Nod2* knockdown has negligible effects on synovial inflammation in mouse OA joints.

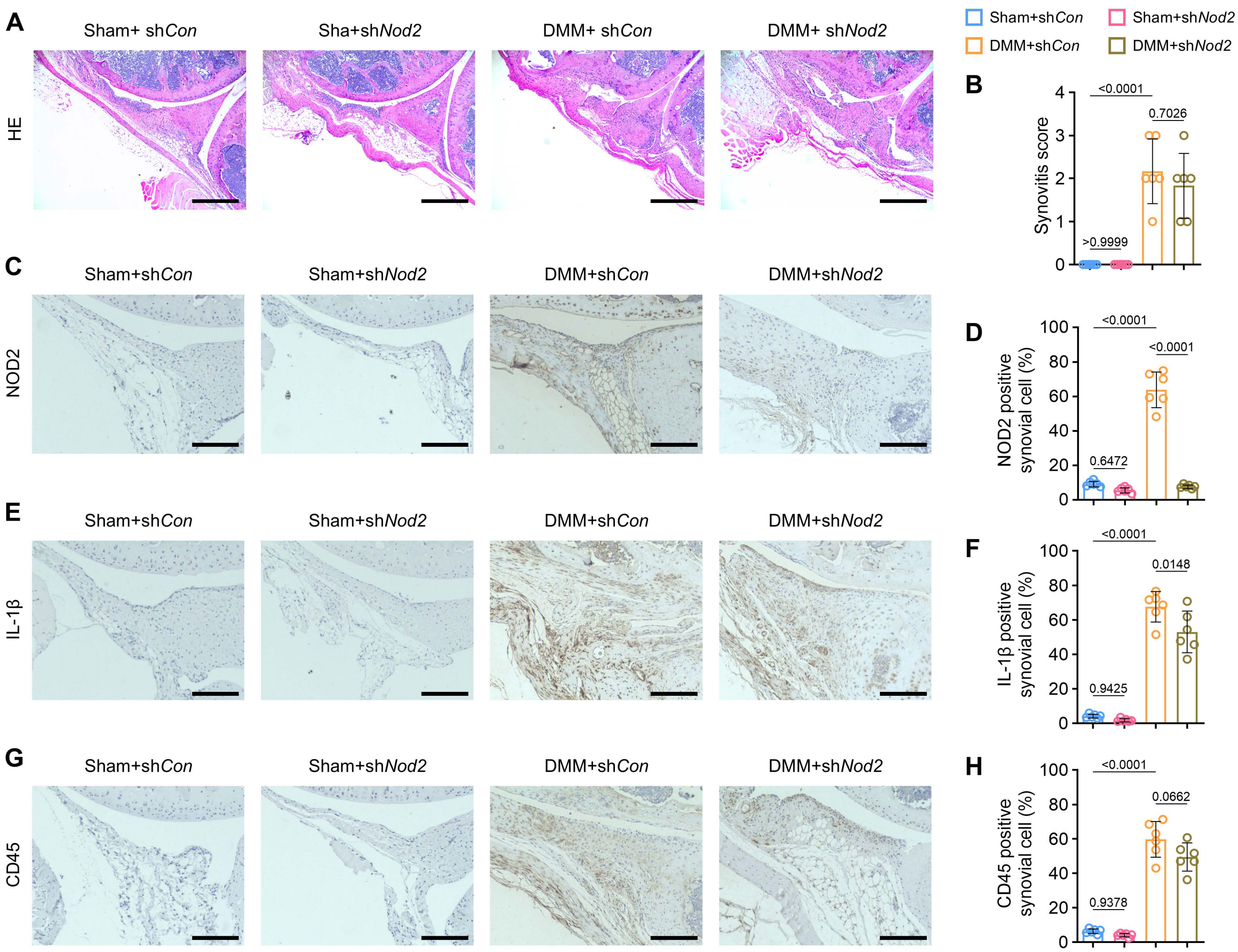

Supplemental Figure 1. Adenovirus-mediated *Nod2* knockdown has negligible effects on synovial inflammation in mouse OA joints. (**A**) Hematoxylin and Eosin (HE) staining of mouse knee joints following adenovirus injection and DMM surgery. Scale bar, 200 μm. (**B**) Quantification of synovitis grading scores based on HE staining. n = 6 mice/group. One-way ANOVA with Tukey's multiple-comparison test. (**C**) IHC staining of knee sections for NOD2. Scale bar: 100 μm. (**D**) Quantification of positively stained synoviocytes for NOD2. n = 6 mice/group. One-way ANOVA with Tukey's multiple-comparison test. (**E**) IHC staining of knee sections for IL-1β. Scale bar: 100 μm. (**F**) Quantification of positively stained synoviocytes for IL-1β. n = 6 mice/group. One-way ANOVA with Tukey's multiple-comparison test. (**G**) IHC staining of knee sections for CD45. Scale bar: 100 μm. (**H**) Quantification of positively stained synoviocytes for CD45. n = 6 mice/group. One-way ANOVA with Tukey's multiple-comparison test. Data are presented as mean ± SD.

Supplemental Figure 2. Post-DMM adenovirus-mediated *Nod2* knockdown attenuates OA development.

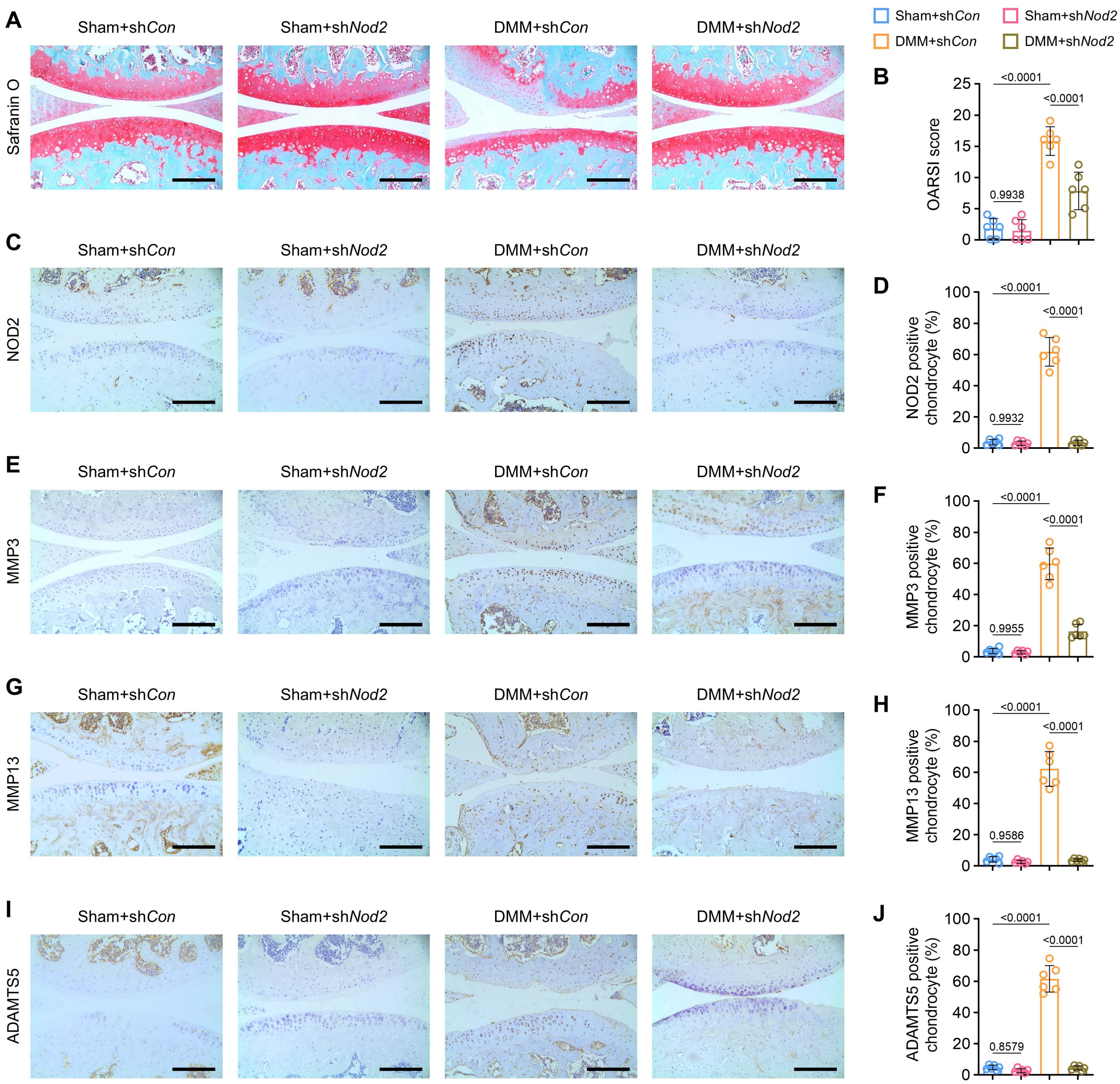

Supplemental Figure 2. Post-DMM adenovirus-mediated *Nod2* knockdown attenuates OA development. (**A**) Safranin O/Fast Green staining of knee joint sections following DMM surgery and *Nod2* knockdown. Scale bar: 100 µm. (**B**) OARSI scores for cartilage degradation based on Safranin O/Fast Green staining. n = 6 mice/group. One-way ANOVA with Tukey's multiple-comparison test. (**C**) IHC staining of knee sections for NOD2. Scale bar: 100 µm. (**D**) Quantification of positively stained chondrocytes for NOD2. n = 6 mice/group. One-way ANOVA with Tukey's multiple-comparison test. (**E-J**) IHC staining of knee sections and quantification of positively stained chondrocytes for MMP3, MMP13, and ADAMTS5. Scale bar: 100 µm. n = 6 mice/group. One-way ANOVA with Tukey's multiple-comparison test. Data are presented as mean ± SD.
