# Supplementary material for: Innate immune sensor NOD2 promotes cartilage degradation and osteoarthritis progression by stabilizing TRAF6 in chondrocytes
Source: JCI Insight. 2026 Aug 24;11(16):e196750. doi: 10.1172/jci.insight.196750 (PMC13502188; doi:10.1172/jci.insight.196750)

Figure 1F

hNOD2
Left

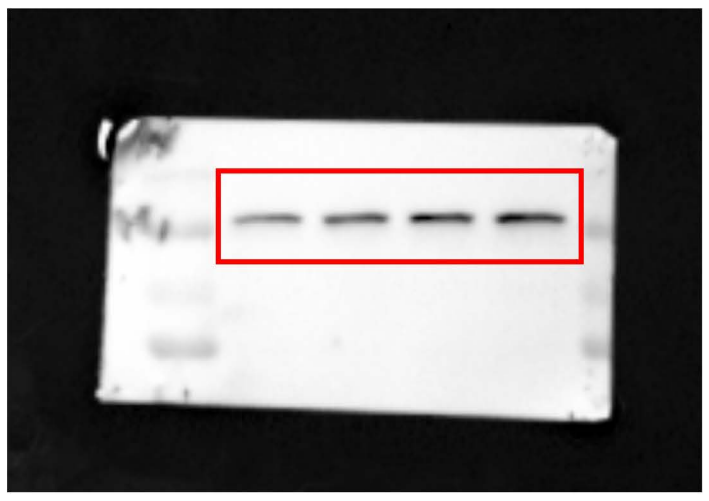

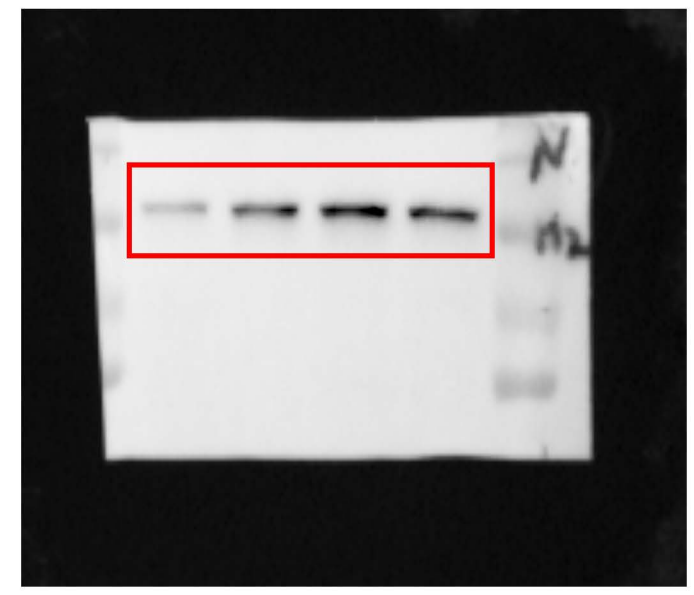

hNOD2
Right

hGAPDH
For
hNOD2
Left

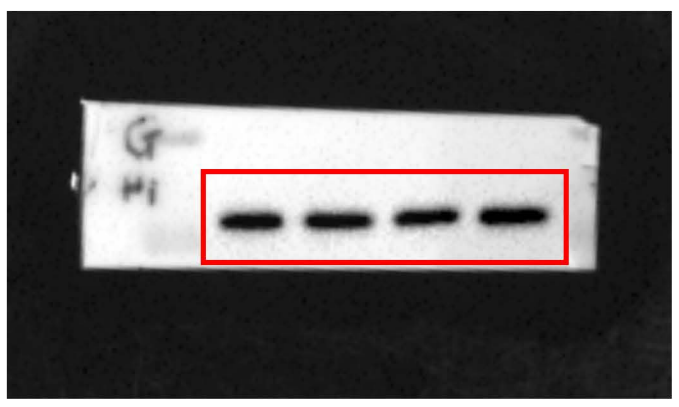

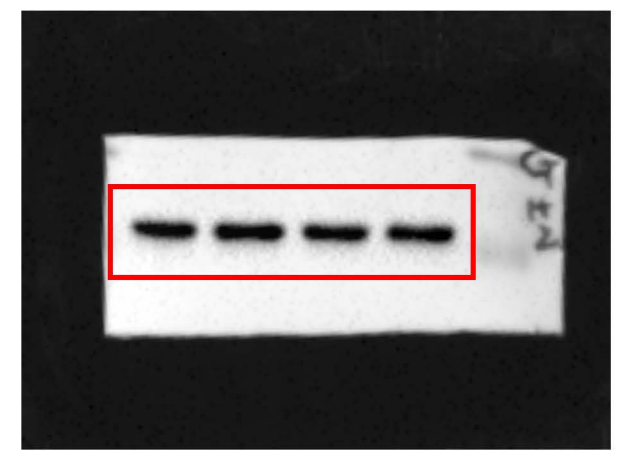

hGAPDH
For
hNOD2
Right

hMMP3
left

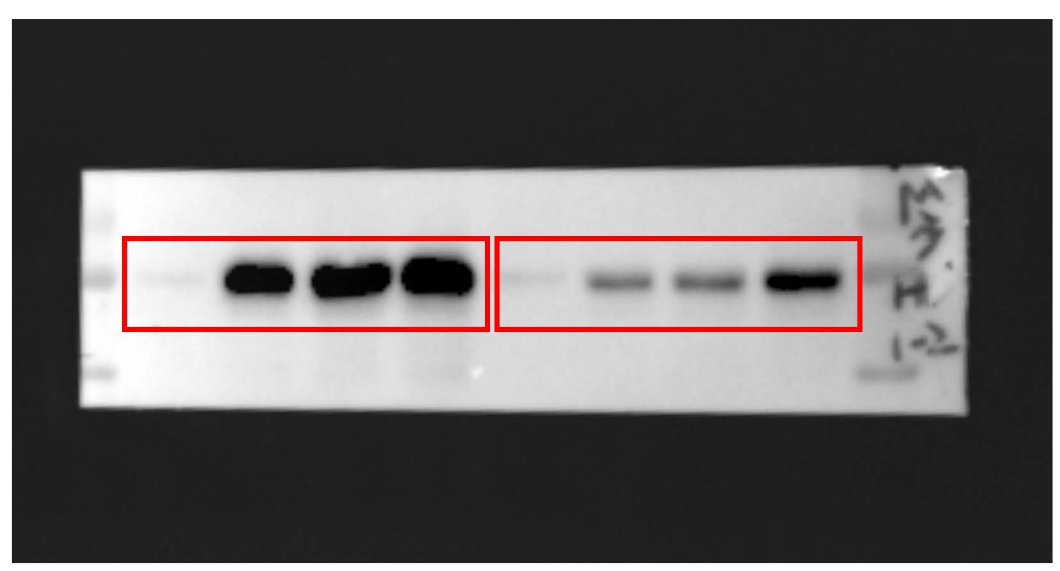

hMMP3
right

hGAPDH
For
hMMP3
left

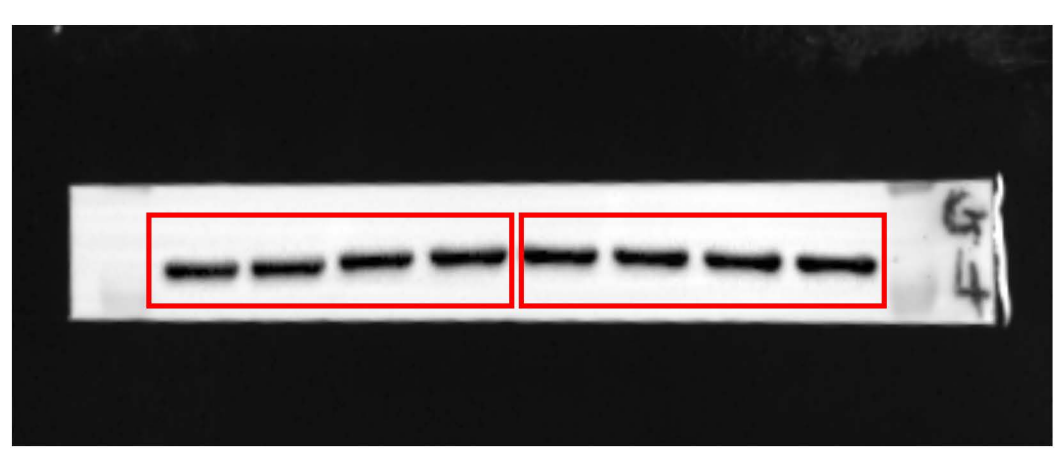

hGAPDH
For
hMMP3
right

Figure 1I

mNOD2

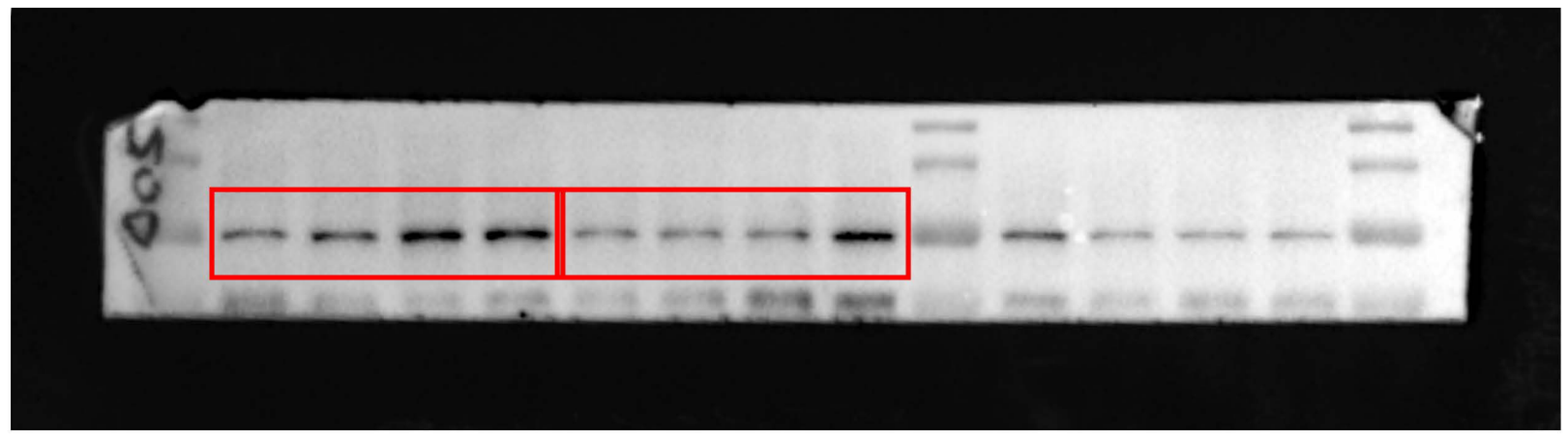

mGAPDH
For
mNOD2

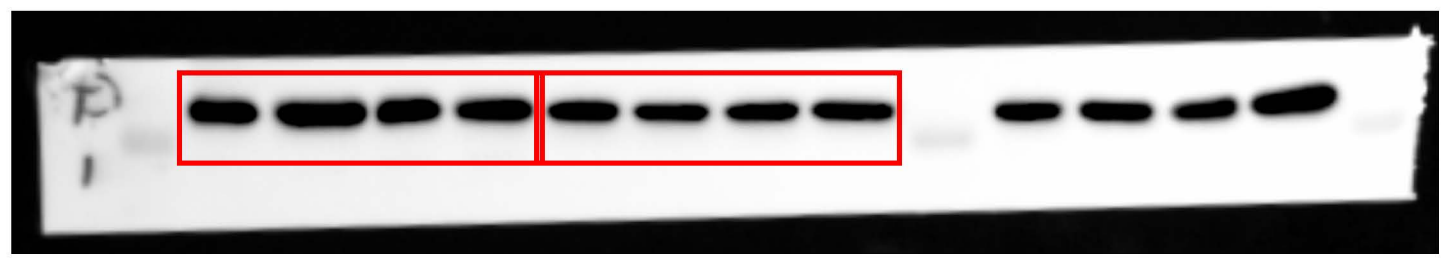

mMMP3

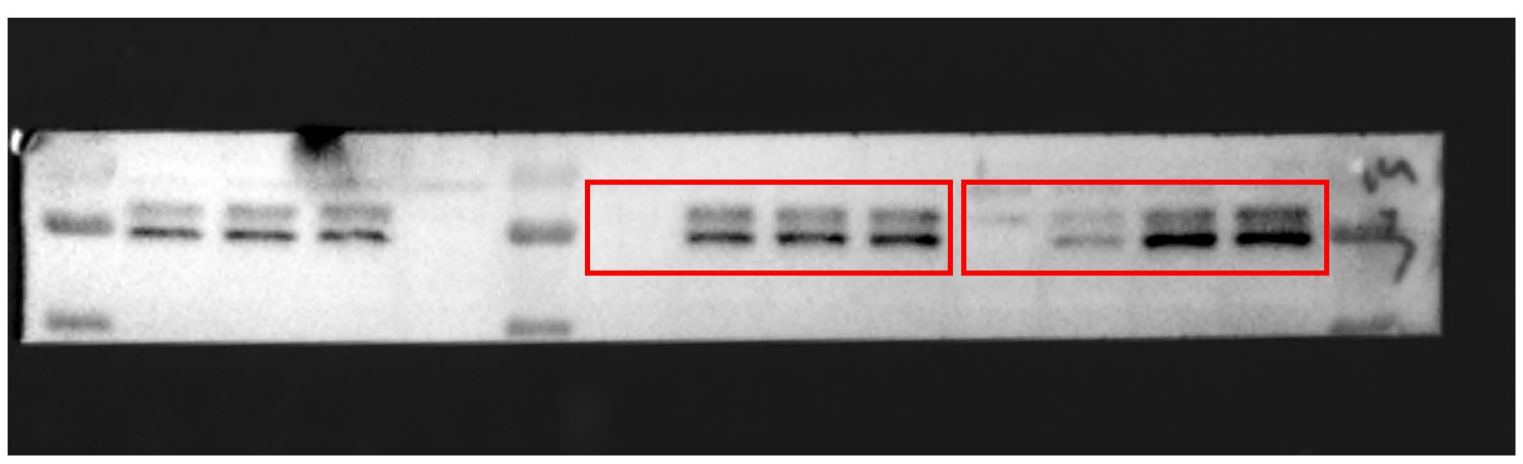

mGAPDH
For
mMMP3

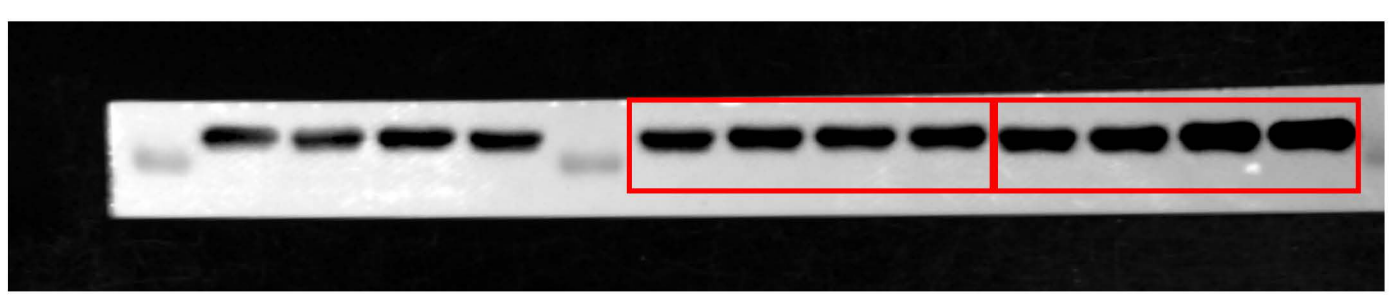

Figure 2B

NOD2

MMP13

ADAMTs5

GAPDH

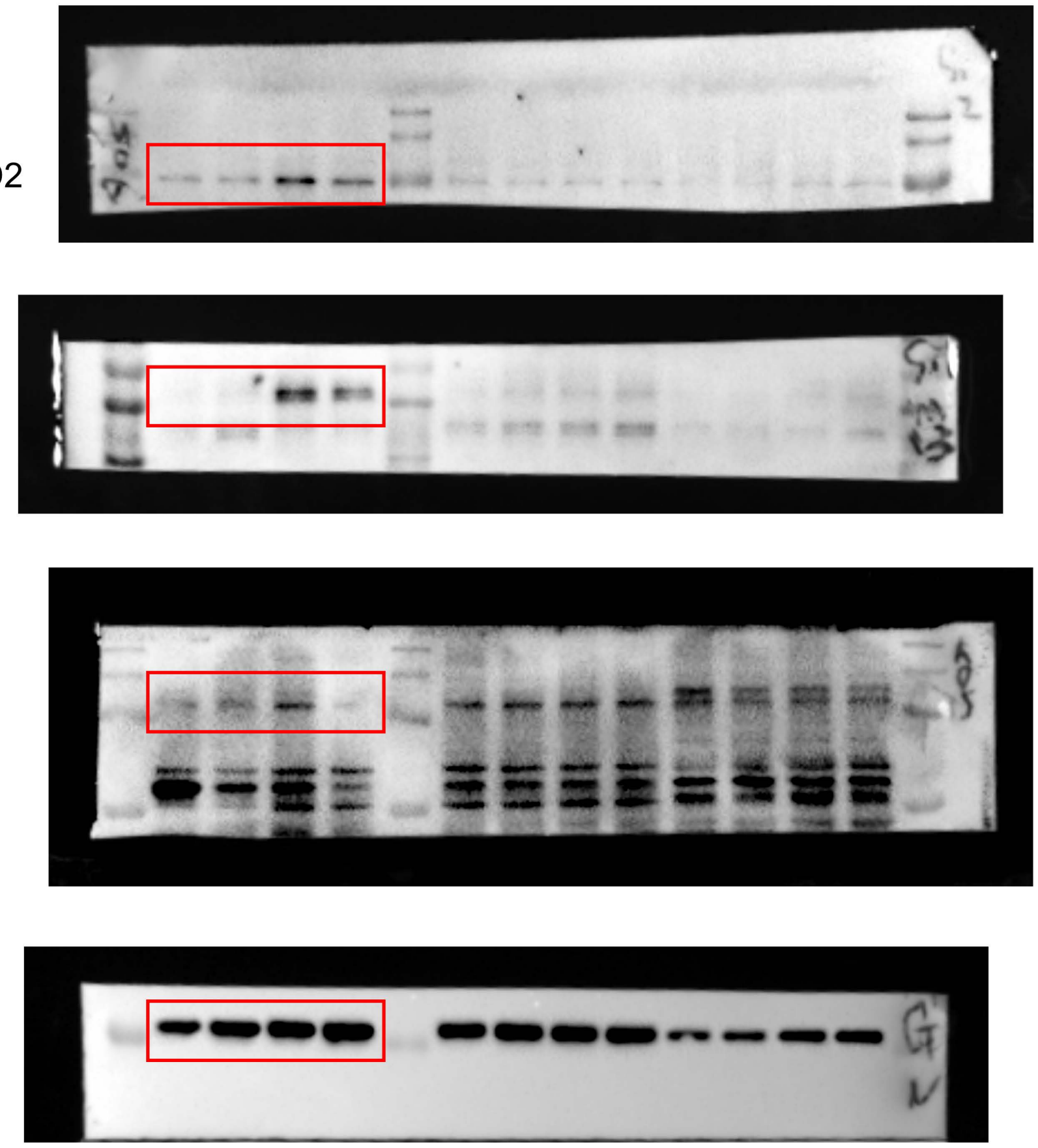

MMP3

GAPDH For MMP3

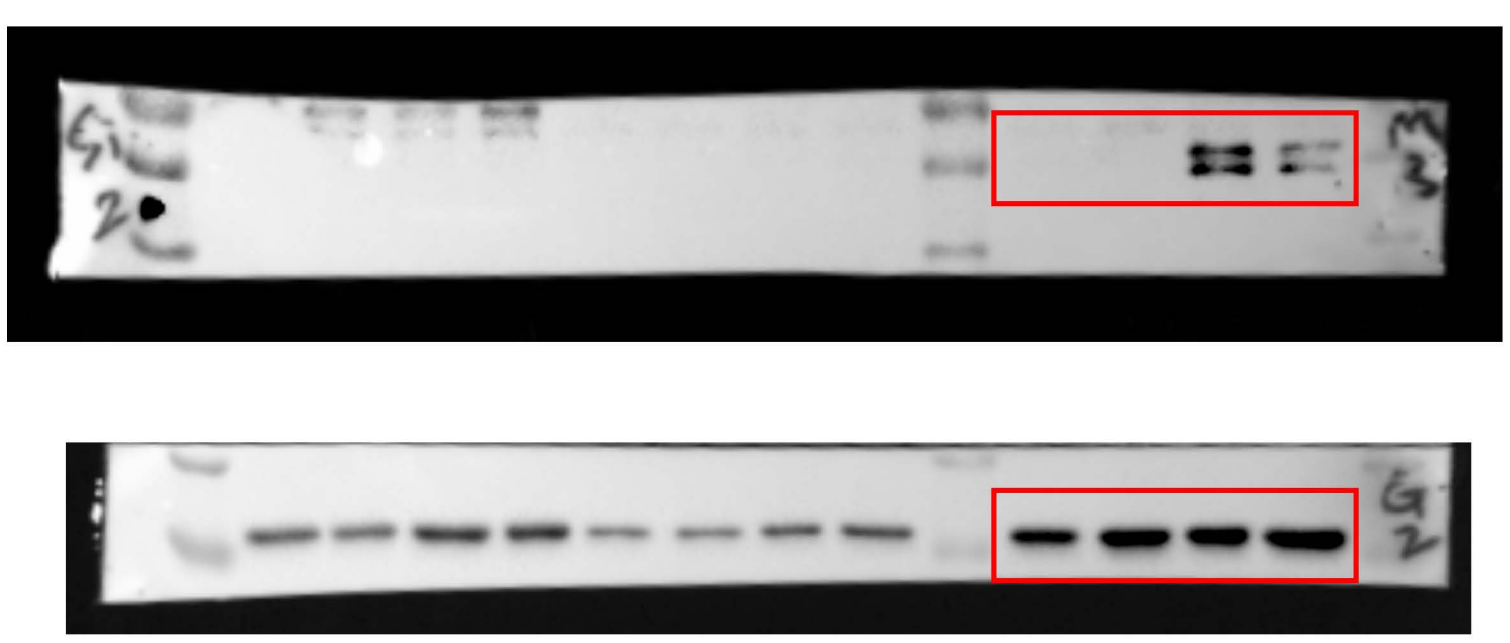

Figure 6A

NOD2

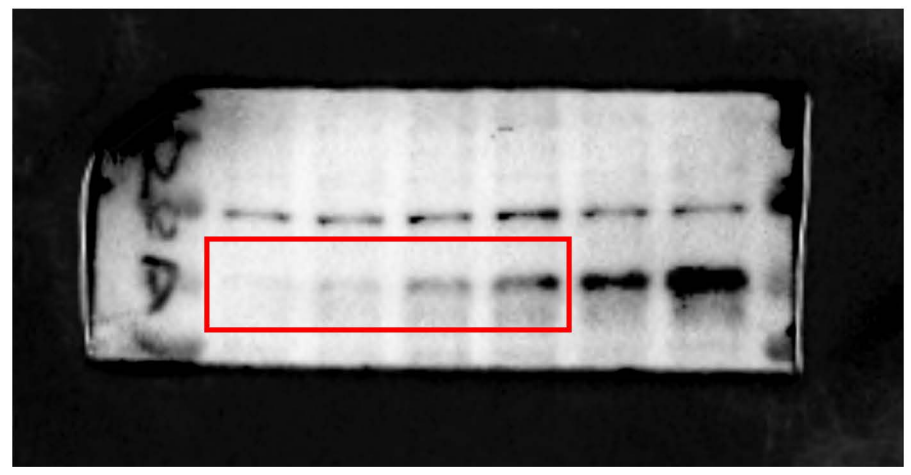

MMP3

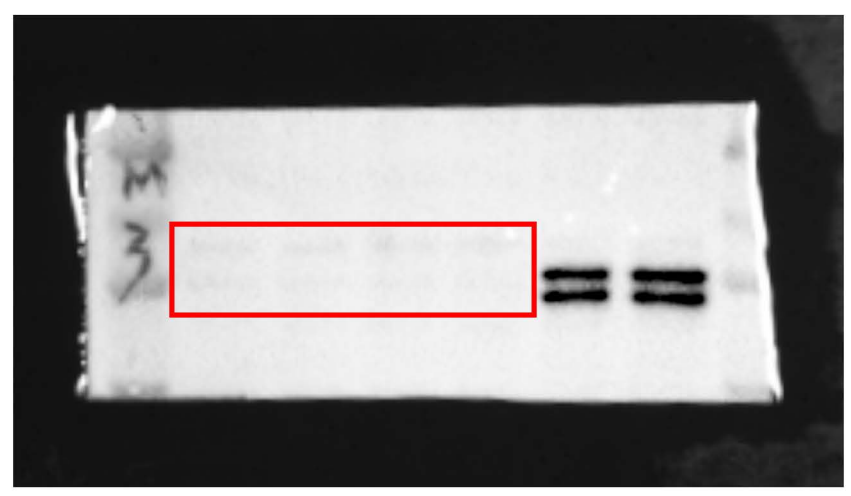

MMP13

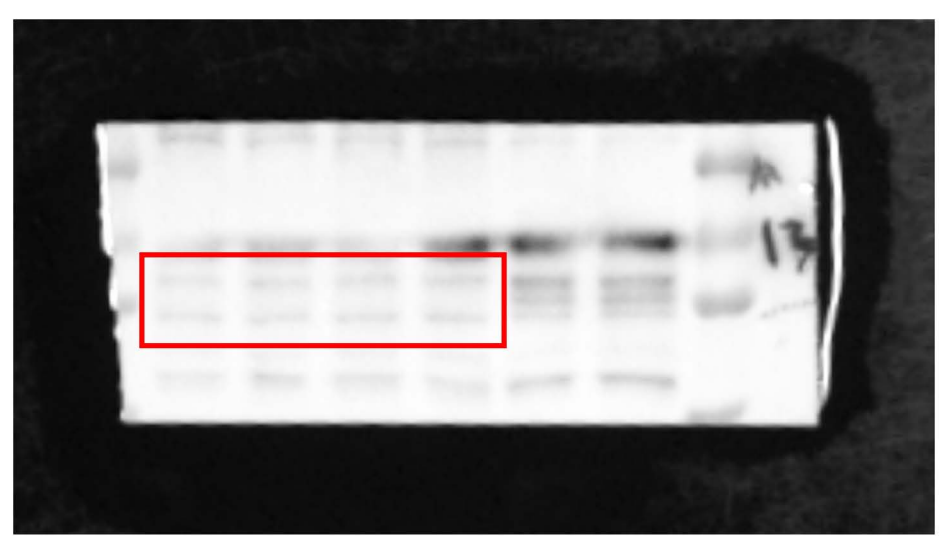

ADAMTs5

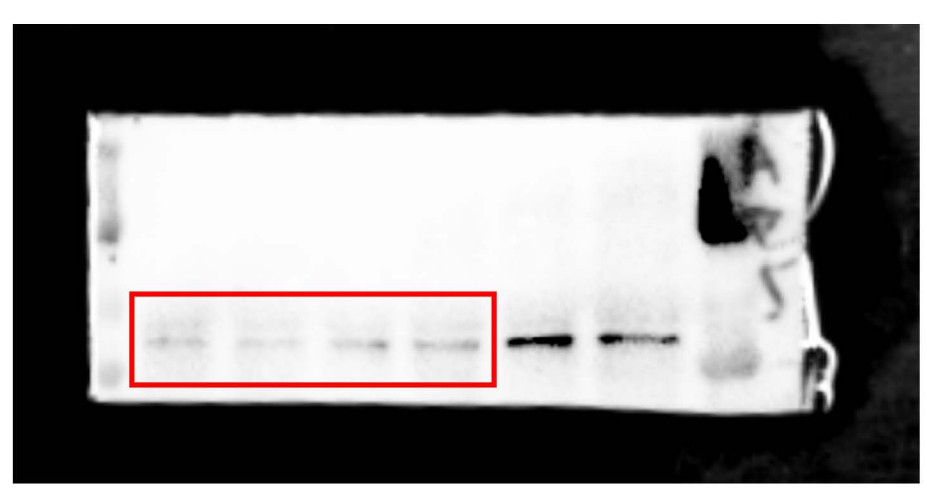

GAPDH

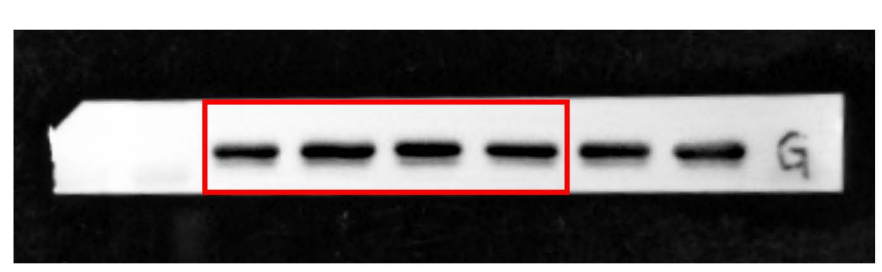

Figure 6I

MMP3

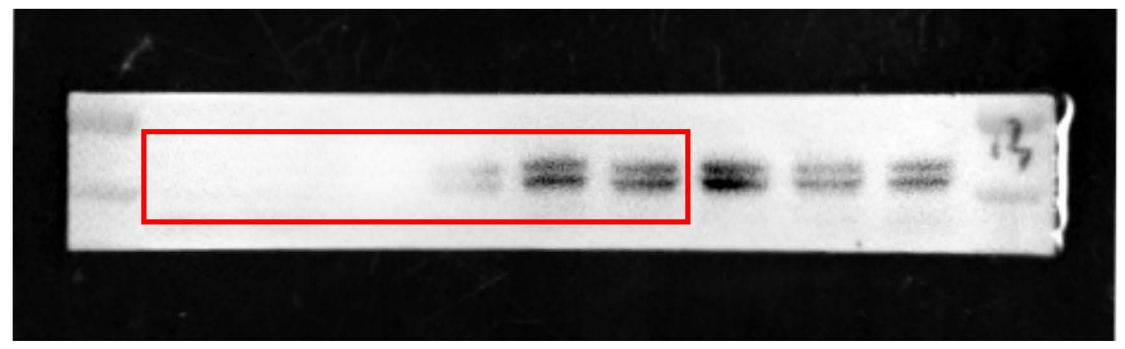

ADAMTs5

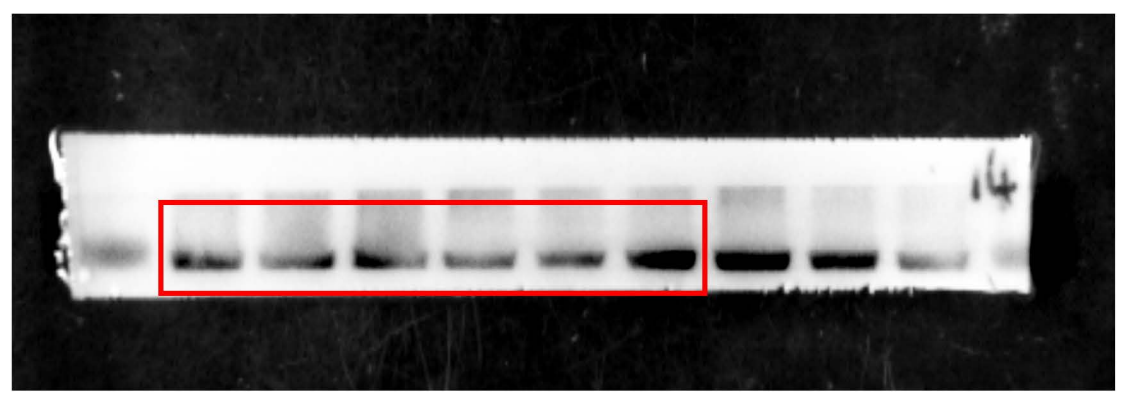

GAPDH

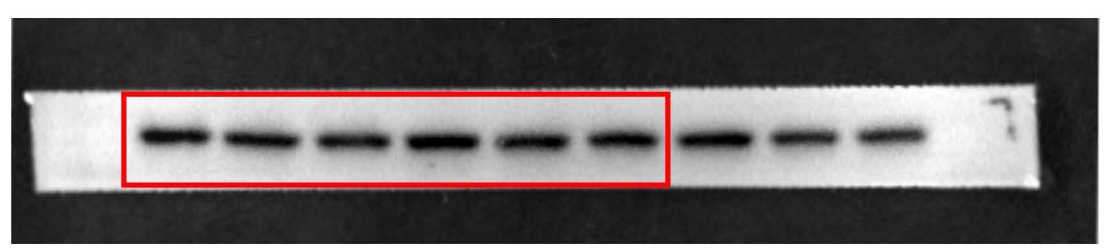

Figure 7B

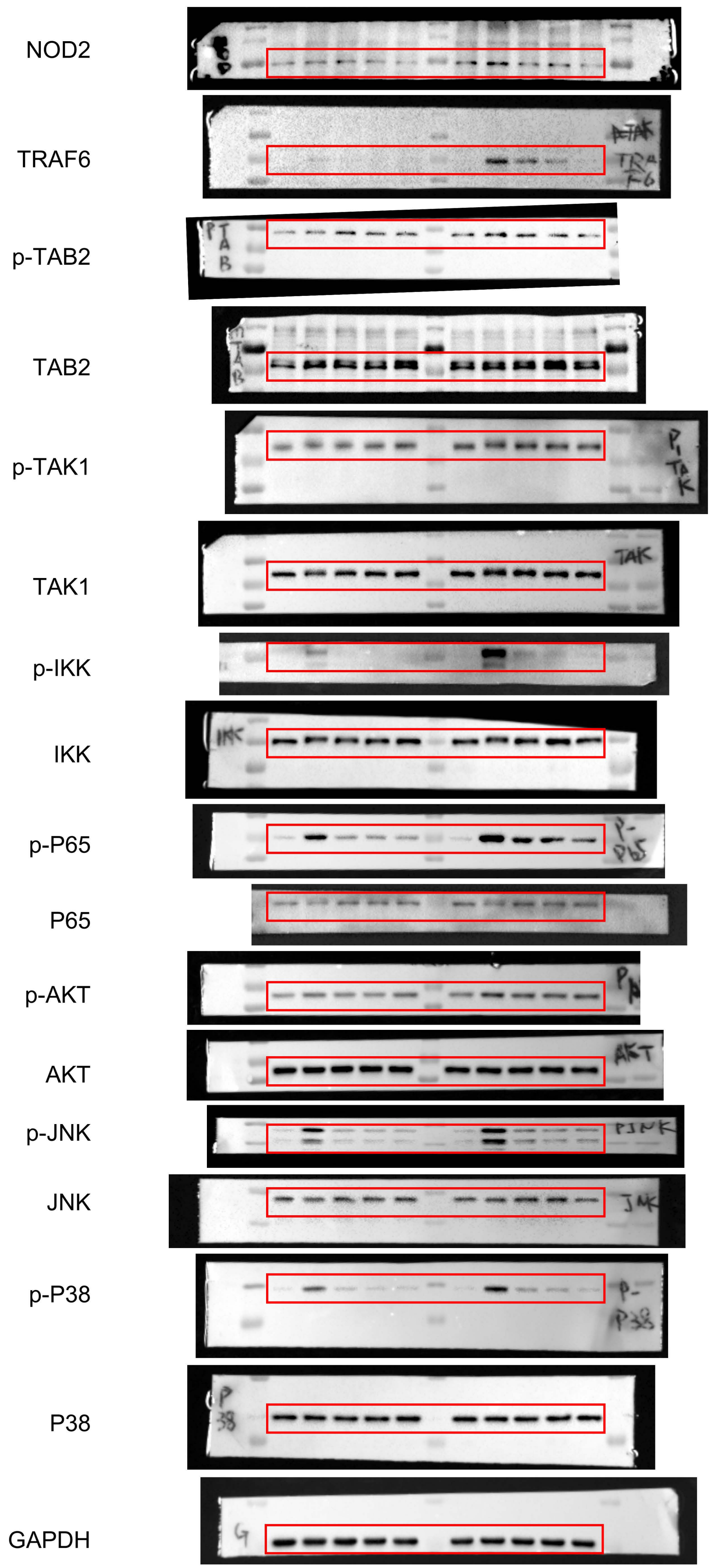
NOD2
TRAF6
p-TAB2
TAB2
p-TAK1
TAK1
p-IKK
IKK
p-P65
P65
p-AKT
AKT
p-JNK
JNK
p-P38
P38
GAPDH

Figure 7D

TRAF6

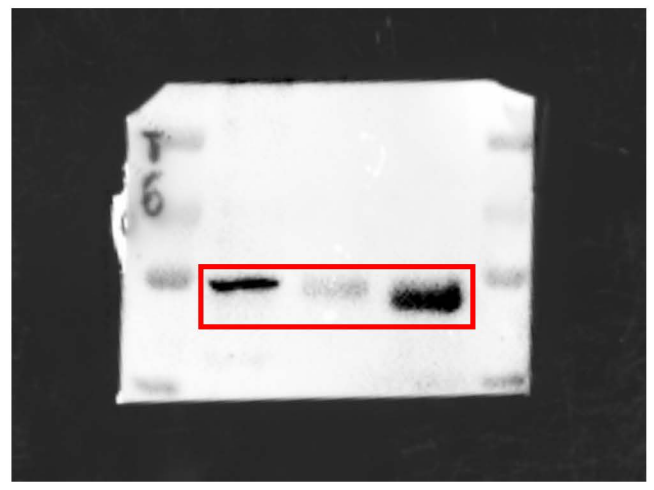

NOD2

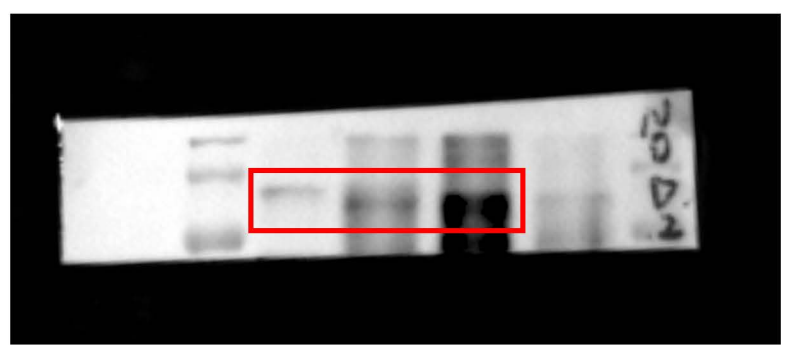

Figure 7E

TRAF6

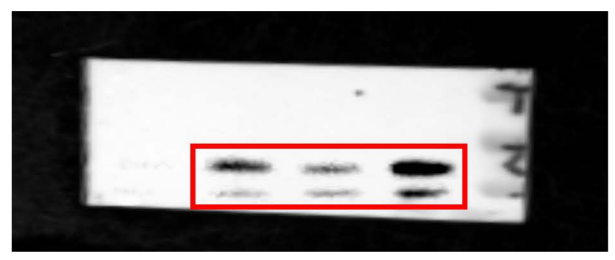

NOD2

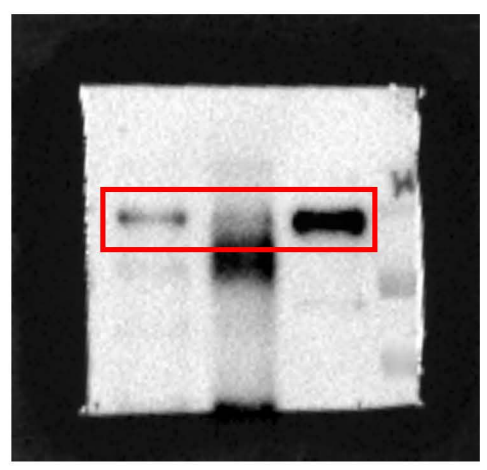

Figure 7F

NOD2

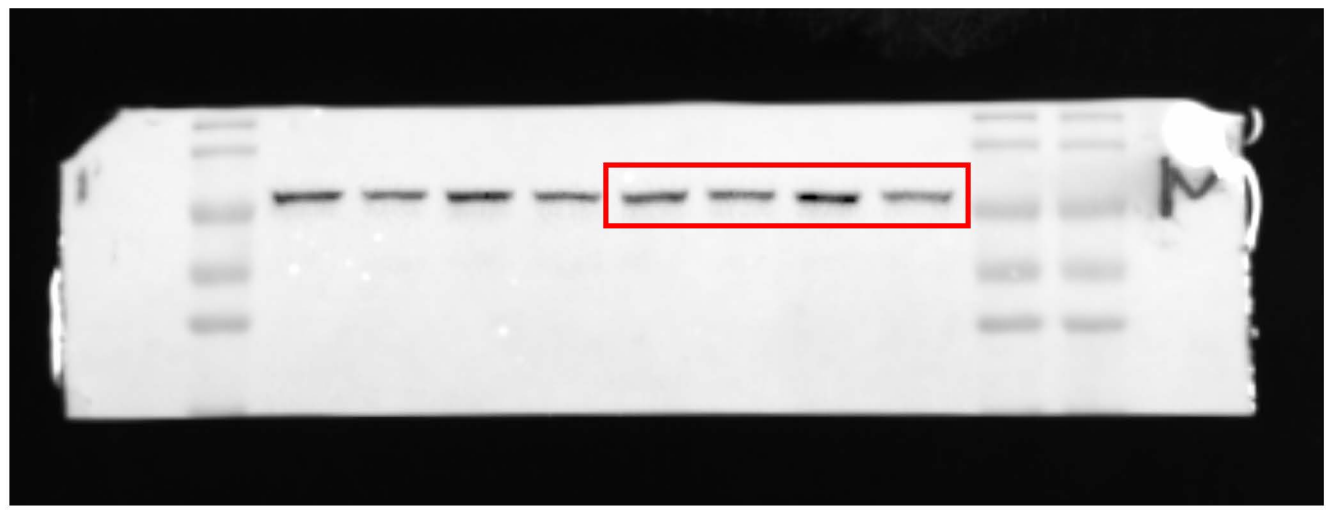

TRAF6

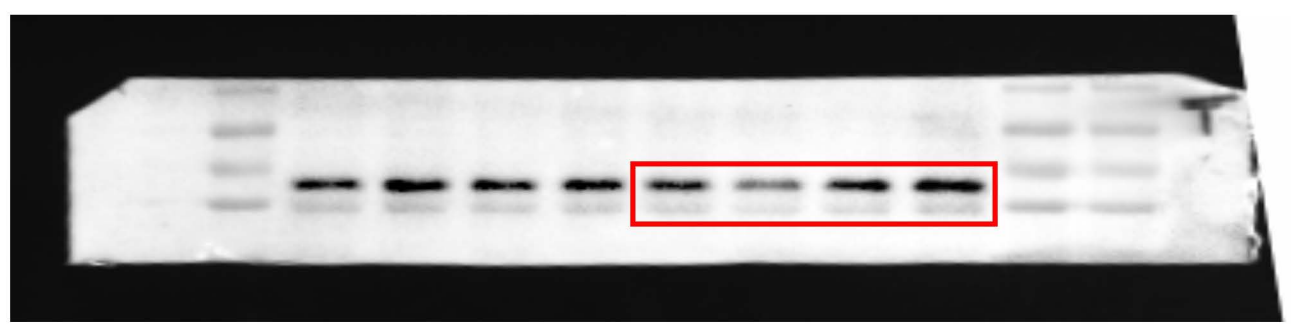

GAPDH

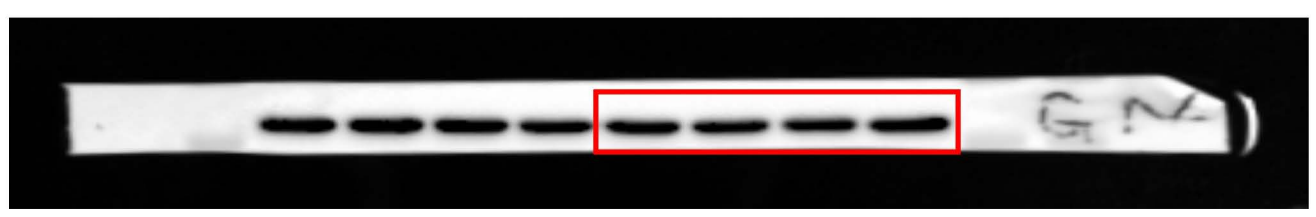

P-TAK1

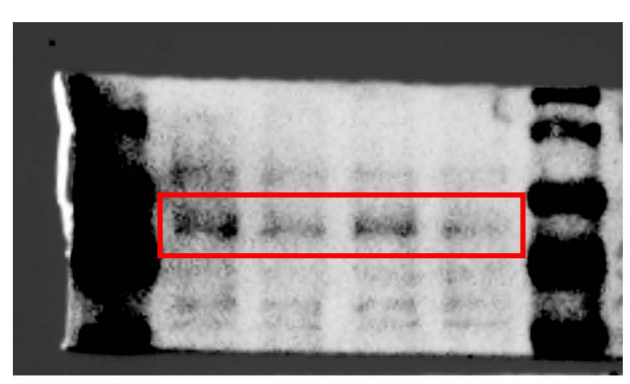

TAK1

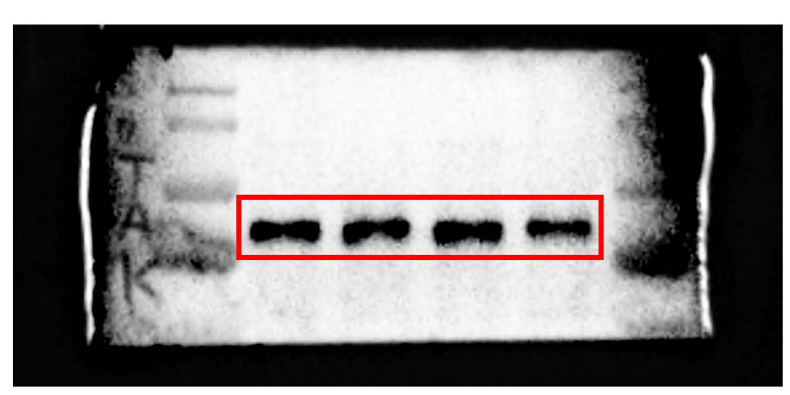

GAPDH

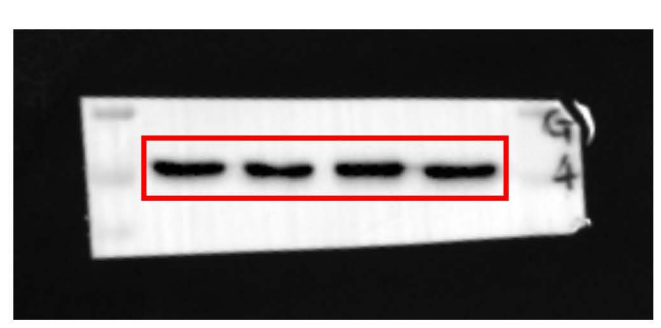

Figure 7G

TRAF6

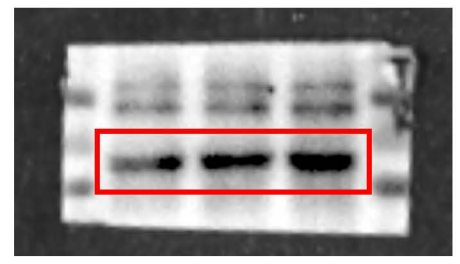

GAPDH

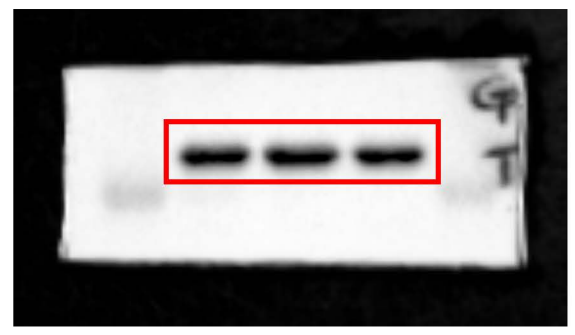

K63-Ub

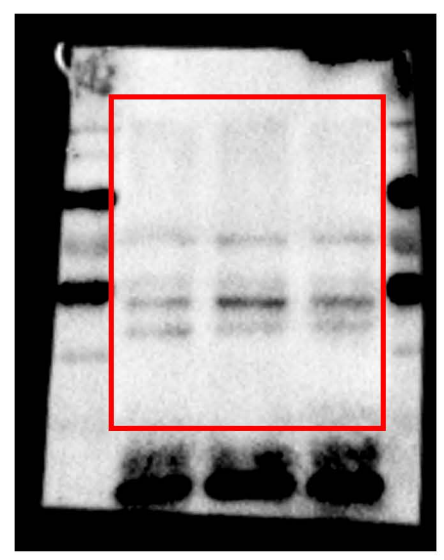

K48-Ub

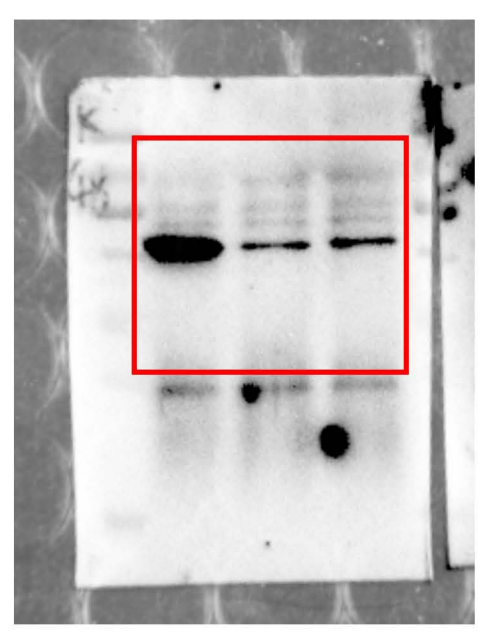

TRAF6

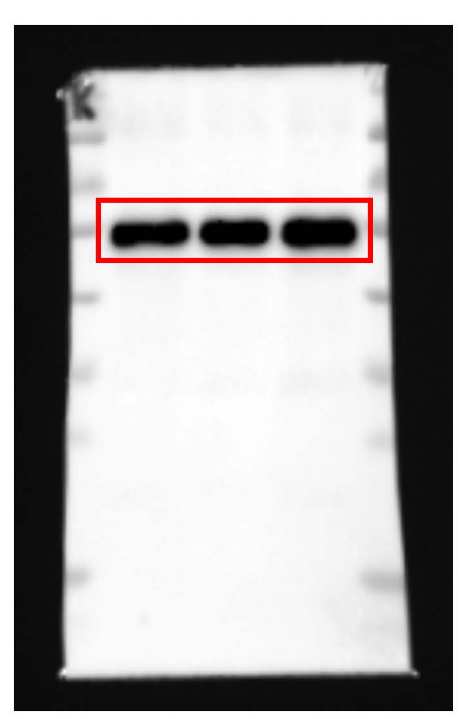

Supplement: Supplementary file 2 [file jciinsight-11-196750-s192.pdf]
